# Upregulation of Siglec-6 induces mitochondrial dysfunction by promoting GPR20 expression in early-onset preeclampsia

**DOI:** 10.1186/s12967-024-05505-z

**Published:** 2024-07-22

**Authors:** Yuanhui Jia, Wenjing Lu, Han Xie, Yifan Sheng, Luyao Wang, Wenqi Lv, Lijun Ling, Jiaqi Dong, Xinrui Jia, Shengyu Wu, Wenqiang Liu, Hao Ying

**Affiliations:** 1 Clinical and Translational Research Center, Department of Obstetrics, Shanghai Key Laboratory of Maternal Fetal Medicine, Shanghai Institute of Maternal-Fetal Medicine and Gynecologic Oncology, Shanghai First Maternity and Infant Hospital, School of Medicine, Tongji University, Shanghai, China; 2 School of Life Sciences and Technology, Tongji University, Shanghai, China

**Keywords:** Preeclampsia, Early-onset preeclampsia, Extravillous trophoblast, Mitochondria, Siglec-6, GPR20

## Abstract

**Background:**

Preeclampsia, especially early-onset preeclampsia (EO-PE), is a pregnancy complication that has serious consequences for the health of both the mother and the fetus. Although abnormal placentation due to mitochondrial dysfunction is speculated to contribute to the development of EO-PE, the underlying mechanisms have yet to be fully elucidated.

**Methods:**

The expression and localization of Siglec-6 in the placenta from normal pregnancies, preterm birth and EO-PE patients were examined by RT-qPCR, Western blot and IHC. Transwell assays were performed to evaluate the effect of Siglec-6 on trophoblast cell migration and invasion. Seahorse experiments were conducted to assess the impact of disrupting Siglec-6 expression on mitochondrial function. Co-IP assay was used to examine the interaction of Siglec-6 with SHP1/SHP2. RNA-seq was employed to investigate the mechanism by which Siglec-6 inhibits mitochondrial function in trophoblast cells.

**Results:**

The expression of Siglec-6 in extravillous trophoblasts is increased in placental tissues from EO-PE patients. Siglec-6 inhibits trophoblast cell migration and invasion and impairs mitochondrial function. Mechanismly, Siglec-6 inhibits the activation of NF-κB by recruiting SHP1/SHP2, leading to increased expression of GPR20. Notably, the importance of GPR20 function downstream of Siglec-6 in trophoblasts is supported by the observation that GPR20 downregulation rescues defects caused by Siglec-6 overexpression. Finally, overexpression of Siglec-6 in the placenta induces a preeclampsia-like phenotype in a pregnant mouse model.

**Conclusions:**

This study indicates that the regulatory pathway Siglec-6/GPR20 has a crucial role in regulating trophoblast mitochondrial function, and we suggest that Siglec-6 and GPR20 could serve as potential markers and targets for the clinical diagnosis and therapy of EO-PE.

**Supplementary Information:**

The online version contains supplementary material available at 10.1186/s12967-024-05505-z.

## Background

Preeclampsia (PE), a condition specific to pregnancy, is diagnosed on the basis of new-onset hypertension (> 20 weeks of gestation) and at least one other associated complication, including proteinuria, maternal organ dysfunction (such as maternal acute kidney injury, liver dysfunction, neurological features, hemolysis or thrombocytopenia) or uteroplacental dysfunction (such as fetal growth restriction or angiogenic imbalance) [1]. PE has a global prevalence of approximately 2–8% and is the main cause of placental abruption, preterm labor, and intrauterine growth restriction of the fetus in both the mother and the baby [2]. PE can be classified based on the timing of clinical signs and symptoms as either early-onset preeclampsia (EO-PE), occurring before 34 weeks of gestation, or late-onset preeclampsia (LO-PE), occurring at or after 34 weeks of gestation [3]. Although abnormal placentation is widely accepted as the origin of EO-PE pathophysiology, this disease, which affects multiple systems, remains inadequately understood. In EO-PE, abnormal extravillous trophoblast (EVT) invasion and deficient spiral artery remodeling lead to reduced blood flow to the placenta. Placental ischemia is significantly associated with the onset of hypertension during pregnancy and seems to be the leading cause of PE. Due to the unknown causes and molecular mechanism of EVT invasion defects and abnormal placentation, induction of labor has been the only available solution for years, leading to a rise in the incidence of preterm birth and neonatal mortality [4].

Mitochondria are cell organelles that serve primarily to provide energy through the synthesis of ATP via oxidative phosphorylation [5]. They contribute to intracellular calcium homeostasis and are critical for several cellular processes, including growth, division, cell migration and invasion [6]. Recently, mitochondrial dysfunction and oxidative stress have been reported as common in preeclamptic placentas [7, 8]. The mitochondria in placental trophoblast cells in PE are characterized by pathological ultrastructural abnormalities such as swelling and reduced cristae or mitochondrial disappearance. Mitochondrial morphology and dysfunction in trophoblasts occur earlier and are more severe in EO-PE than in LO-PE [8]. Trophoblast mitochondrial morphology and dysfunction play a crucial role in the development of PE, but the regulatory mechanisms and causes of abnormalities have yet to be determined.

Siglec-6 belongs to the family of sialic acid-binding immunoglobulin-like lectins (Siglecs), which are transmembrane receptors [9]. Siglec-6 expression is specific to primates and is highly expressed in the human placenta. Additionally, PE is a disease that only affects humans [10, 11]. Siglec-6 is expressed in placental syncytiotrophoblasts, cytotrophoblasts and EVTs [12]. Studies report that Siglec-6 expression is significantly upregulated in the placental tissues of PE. Moreover, the expression of Siglec-6 in placental tissues of EO-PE is significantly higher than that of LO-PE [13]. Additionally, various members of the Siglec family have been found to play roles in the regulation of mitochondrial oxidative phosphorylation and energy metabolism [14, 15]. However, no study has reported whether Siglec-6 plays a role in the development of PE by regulating mitochondrial morphology and function.

In this study, we compared the expression and localization of Siglec-6 in the EO-PE placenta with that in placentas from normal pregnancies and preterm births. The effects of Siglec-6 on trophoblast migration, invasion and mitochondrial function and the mediating mechanisms were subsequently explored. Finally, we infected mouse blastocysts with lenti-Siglec-6, achieved specific expression of Siglec-6 in the murine placenta, established a novel mouse model, and determined the critical role of Siglec-6 in the pathogenesis of PE.

## Methods

### Tissue collection

This study was approved by the Ethics Committee of Tongji university (Approval ID: 2021tjdx090). We collected placental tissues from a total of 45 specimens from the third-trimester placenta of EO-PE patients, normal and preterm birth (healthy pregnant women who have a spontaneous preterm birth) from February 2020 to July 2022. The tissues were obtained immediately (< 30 min) after delivery by caesarean section. The diagnostic criteria for PE were in accordance with the 2019 ACOG Bulletin. We also noted normal blood pressure in both normal and preterm birth groups, and the absence of pregnancy complications and comorbidities. We excluded pregnant women with fetal malformations, infections, multiple pregnancies, gestational diabetes and autoimmune diseases. All of the pregnant women underwent cesarean sections in the third trimester, and all participants signed an informed consent form that allowed us to conduct research with the obtained tissues. Clinical information on pregnant women is shown in supplementary Table 1.

### Cell culture and treatment

Immortalized HTR-8/SVneo cells and JAR cells were kindly donated by the Obstetrics and Gynecology Hospital of Fudan University. The HTR-8/SVneo and JAR cells, with a passage number of approximately 20–30, were cultured using DMEM/F12 medium (HyClone, USA) which contained 10% fetal bovine serum (FBS, FBSAD-01011-500), 100 µg/ml streptomycin and 100 U/ml penicillin (Gibco, USA) at 37 ℃ in a humidified 5% CO_2_ incubator. Lipofectamine 3000 reagent (Invitrogen) was used for HTR-8/SVneo and JAR transient transfection. HTR-8/SVneo and JAR cells were seeded into a 6-well plate. To suppress the expression of Siglec-6, GPR20 and Leptin R, the cells were transfected with 3.2 µg of siRNAs by 5 µL Lipofectamine 3000 per well when they reached about 30% confluence. The sequence of the siRNAs is listed in Table S1. For Siglec-6 overexpression, cells were infected by using the lentiviral vector pHBLV-CMV-MCS-EF1-ZsGreen1-T2A-puro (HANBIO Shanghai, China) and next selected with 1 µg/ml puromycin (Sigma). The transfection efficiency was determined by quantitative real-time PCR and Western blot.

### RNA isolation, reverse transcription, and quantitative real-time PCR

Total RNA from placental tissues and HTR-8/SVneo/JAR cells was isolated using RNAiso Plus (Takara) and reverse transcribed into cDNA using Prime-Script™ RT Master Mix (Takara) according to the manufacturer’s instructions. Quantitative PCR was carried out by using SYBR Premix ExTaq™ (Takara) on the StepOne Plus™ Real-Time PCR system (Applied Biosystems, Inc.) according to the manufacturer’s instructions. The PCR primer sequences used in our study are listed in supplementary Table 2. The relative levels of mRNA were analyzed using the 2^−ΔΔCT^ method and normalized to β-actin.

### Western blot

Proteins were extracted from specimens and cultured cells using RIPA buffer (Beyotime, P0012B). In order to inhibit both proteases and phosphatases, the complete ULTRA tablets (Roche, Basel, Switzerland) were combined with the PhosSTOP EASYpack tablets (Roche, Basel, Switzerland). Protein concentrations were determined with the BCA assay (Thermo Fisher Scientific, 23,227). Each sample containing 200 µg of protein was mixed with loading buffer (Beyotime, P0015L) and separated in 4-20% SDS-PAGE gels (Beyotime, P0524M). The samples were subsequently transferred onto polyvinylidene difluoride (PVDF) membranes (Millipore, ISEQ00010). Following the blockade of the membranes with 5% non-fat milk in TBST, the membranes were incubated with primary antibodies at 4℃ overnight and were subsequently probed with secondary antibodies conjugated with horseradish peroxidase for 1 h at room temperature. All antibodies used in this study were listed in Table S3. All bands were imaged using enhanced chemiluminescence reagent (Millipore, WBKLs0500) with an Imaging System from Tanon. β-actin was employed as an endogenous control for loading.

### Immunohistochemistry (IHC)

Placental tissues were fixed in 4% paraformaldehyde, embedded in paraffin, sectioned (5 μm), and deparaffinized and hydrated. After the antigen retrieval according to antibody instructions, endogenous peroxidase activity was blocked with 3% hydrogen peroxide/methyl alcohol for 10 min. Nonspecific binding sites were blocked with 5% BSA for 30 min, after which the sections were incubated with the primary antibody overnight at 4 °C. The primary antibody information is listed in Table S3. After incubation with the primary antibody, the sections were treated with the secondary antibody (abcam, ab6721) at room temperature for 1 h, followed by treatment with DAB reagent. Slices were counterstained with hematoxylin, and DAB positivity was assessed using Image-Pro Plus 6.0 on each section.

### Migration and invasion assays

The transfected cells were suspended in 200 µL of serum-free medium and seeded into the upper chamber (8 μm pore size, 3422, Corning, USA) with a density of 5 × 10^4^ cells per well. The lower chamber was filled with 500 µL of DMEM/F12 medium, which had been supplemented with 10% FBS. Recombinant human leptin protein (R&D, 398-LP-01 M) was added to both the upper and lower chambers at concentrations of 100 ng/mL or 200 ng/mL. The plate was incubated at 37 °C with 5% CO_2_ for 24 h. After that, the cells were fixed with 4% paraformaldehyde for 20 min and stained with 0.2% crystal violet. Non-invaded cells that on the inner side of the inserts were removed using cotton swabs. The invasion assay was performed in a similar manner, but the membranes were pre-coated with 100 µL of Matrigel (400 µg/ml, BD Science) in serum-free medium for 60 min at 37 °C and 1 × 10^5^ cells were seeded in the upper chamber of the chamber. A microscope was used to capture and count the number of cells present in three random fields.

### Cell proliferation assay

Cells were seeded in 96-well plates at a density of 5 × 10^3^ cells per well and allowed to proliferate for 24 h. Cell proliferation was then assessed using the Alexa Fluor 555 kit (CX003, Epizyme, Shanghai, China) with 5-Ethynyl-2’-deoxyuridine (EdU), according to the manufacturer’s instructions. After photography under fluorescent microscopy in 5 different fields of view, the cells were counted using ImageJ.

### Co-immunoprecipitation (Co-IP)

HTR-8/SVneo cells were transfected with an expression plasmid encoding a Flag-tagged Siglec-6 cDNA, then lysed with IP lysis buffer (P0013, Beyotime, Haimen, China). The lysates were incubated with anti-flag, anti-SHP1 and anti-SHP2 antibodies overnight at 4 °C. The precipitated with the antibody protein complex using protein A/G beads (Thermo Fisher Scientific, Waltham, MA). The immunoprecipitates were washed five times with IP lysis buffer and then subjected to Western blotting analysis.

### Chromatin immunoprecipitation (ChIP)

1 × 10^7^ cells was cross-linked with 1% formaldehyde for 15 min at room temperature and quenched with 0.125 M glycine. The cells were incubated in a lysis buffer (150 mM NaCl, 25 mM Tris pH 7.5, 1% Triton X-100, 0.1% SDS, 0.5% deoxycholate) containing protease inhibitors. The lysed sample was subjected to sonication, resulting in the generation of DNA fragments with an approximate length of 200–1000 bp. Then, the target protein-DNA complexes were immunoprecipitated with 5 µg of anti-NF-κB/p65 antibody (Cell Signaling Tech, 8242). Following dissociation from the immune complexes, the immunoprecipitated DNAs were quantified by qPCR and normalized against the genomic DNA input prepared before immunoprecipitation. The sequence of the primer is listed in Table S3.

### Double luciferase reporter assay

PCR amplification of the human GPR20 promoter region (− 2000 to + 86 relative to the transcription start site) generated GPR20 promoter constructs. These constructs were then subcloned into the pGL3-basic vector (Promega, Madison, WI, USA), which contains a firefly luciferase gene. Then 293T cells were transiently transfected with a pGL3-GPR20 plasmid, pLVX-p65 and a pRL-TK plasmid that contains a Renilla luciferase gene as an internal control. After 48 h of transfection, the activities of luciferases were measured using a Dual-Luciferase Reporter System (Promega, Madison, WI, USA) according to the manufacturer’s instructions, activities of luciferases were recorded by a SpectraMax M5 spectrophotometer (Molecular Devices). Relative luciferase activity in each well was normalized to the ratio of firefly luminescence to Renilla luminescence.

### Enzyme-linked immunosorbent assay (ELISA)

The HTR-8/SVneo vector and HTR-8/SVneo Siglec-6 overexpression cells were seeded in 24-well plates at 2 × 10^5^ cells per well. After 48 h, adherent cells were directly lysed in 200 µL of 0.1 M HCl, and the intracellular c-AMP levels were measured using a c-AMP assay Kit (ab65355, Abcam, USA) according to the manufacturer’s instructions.

### RNA sequencing

Total RNA was isolated from the HTR-8/SVneo vector and HTR-8/SVneo Siglec-6 overexpression cells using TRIzol reagent (Life Technologies, Carlsbad, CA) and each group had three biological repeats. A Nanodrop ND-2000 spectrophotometer (Thermo Fisher Scientific, Waltham, MA) was used to assess RNA concentration and purity. RNA purity and integrity were measured via 1.5% agarose gel electrophoresis. The Agilent 2100 Bioanalyzer (Agilent Technologies, Santa Clara, CA) was used to confirm an RNA integrity number value > 7.0.

A sequencing library was produced using the TruSeq RNA preparation kit (Illumina, San Diego, CA) according to the manufacturer’s instructions. Briefly, magnetic beads with Oligo (dT) were used to purify mRNA, which was then fragmented into short fragments of approximately 200 bp. cDNA was synthesized and purified. The Qubit 2.0 instrument (Thermo Fisher Scientific, Waltham, MA) was used to assess the concentration of cDNA. The length of library fragments was determined using the Agilent 2100 Bioanalyzer. Sequencing was carried out using an Illumina HiSeq 2500 platform (Illumina, San Diego, CA), resulting in the production of 125 bp paired-end reads.

The clean reads were aligned to the human genome (GRCh 38) using HISAT2. The FPKM value of each transcript was calculated using Cufflinks. The DESeq (2012) R package was employed to identify differentially expressed genes (DEGs), whereby a P value < 0.05 and |log2FoldChange| > 1 were set as the threshold for significant differential expression.

### Mitochondrial respiratory chain complex III and complex IV activities

The activities of mitochondrial complex III and IV were measured using the Mitochondrial complex III and IV Activity Assay Kit (BC3245, BC0945, Solarbio, Beijing, China). According to the manufacturer’s instructions, HTR-8/SVneo Siglec-6 knockdown and overexpression cells were harvested and the protein concentration of cell lysates was also determined using the BCA assay (23,225, Thermo Fisher, USA). The absorbance (550 nm) was measured at specific optical densities using a SpectraMax M5 spectrophotometer (Molecular Devices).

### ROS levels

HTR-8/SVneo Siglec-6 knockdown and overexpression cells were seeded in 96-well plates and after 24 h the cells were incubated with the master reaction mix for 1 h at 37 °C, then the intracellular ROS levels were analyzed using the Fluorometric Intracellular ROS Kit (MAK145, Sigma, USA) according the provided instructions. Fluorescence was measured at an excitation wavelength of 520 nm and an emission wavelength of 605 nm using a microplate reader.

### Mitochondrial membrane potential

HTR-8/SVneo Siglec-6 knockdown and overexpression cells were seeded the day before in 48-well plates at 5 × 10^4^ per well. The cells were incubated for 10 min at 37 °C under 5% CO_2_ with PBS for the experimental group or with 10 µM carbonyl cyanide m-chlorophenyl hydrazone (CCCP) for the positive control. Mitochondrial membrane potential was measured using the TMRE assay kit (C2001S, Beyotime, Shanghai, China). After treating and staining, cells were analyzed by flow cytometry following the manufacturer’s instructions.

### Transmission electron microscopy (TEM)

Human placenta and mouse kidney tissues were fixed overnight at 4 °C with 2.5% glutaraldehyde followed by 1% osmium tetroxide fixation for 2 h. Tissues were then embedded, sectioned, stained, and visualized under TEM [16]. The images were captured using a HT7700 transmission electron microscope (HITACHI, Japan). The image analysis software ImageJ was used to determine the area of the placental mitochondria.

### Oxygen consumption rate (OCR)

OCR was assessed using the Sea-horse XFe96 Extracellular Flux Analyzer (Agilent, CA) to analyze mitochondrial respiratory function. The seeded HTR-8/SVneo and JAR cells were treated and incubated for 24 h in XFe96 Cell Culture Microplates at a density of 2 × 10^4^, at 37 °C. To perform the mitochondrial stress test, the cultured medium was supplemented with 0.5 µM oligomycin, 0.125 µM FCCP and 0.5 µM rotenone plus 0.5 µM antimycin A.

### Animals study

#### Mice and lentiviral transduction

6–8 weeks-old adult ICR mice were procured from Beijing Vital River Laboratory Animal Technology Co. and were maintained under specific pathogen-free conditions (room temperature: 22 ± 2 °C; relative humidity: 55 ± 10%) on a light: dark cycle of 12:12 h of artifical light (lights on from 6:00 a.m. to 6:00 p.m.). The mice were fed with a normal diet which were purchased from Shanghai Puluteng Biological Technology Co., Ltd (no. P1101F). All animal studies were performed in compliance with the experimental animals use regulations issued by the National Research Institute and were approved by Tongji University. All operations were in accordance with the requirements of animal ethics. The mice were divided into two groups: control and overexpression groups, comprising 11 mice labelled as vector per group and 11 mice labelled as Siglec-6-OE per group. The control group was treated with empty lentivirus infected blastocysts while the Siglec-6-OE group was treated with Siglec-6 overexpression lentivirus infected blastocysts.

#### Measurement of blood pressure and urinary protein

Blood pressure measurements were taken between 09:00 a.m. and 12:00 p.m. using a CODA noninvasive plethysmography blood pressure transducer (CODA HT8, Kent Scientific). To ensure at least 5 sets of data were available, systolic and diastolic blood pressure were recorded more than 10 times for an average of 10 min. Metabolic cages were used to collect urine from each mouse separately at GD10, GD12, GD14, GD16, and GD18 (09:00 a.m. to 09:00 a.m. the following day). Proteinuria concentration was measured using a BCA protein assay kit (23,225, Thermo Fisher, USA), with urinary protein calculated as proteinuria concentration multiplied by urine volume.

#### Histology

Mice were executed at GD18.5d, and the fetuses and attached placenta were promptly collected and weighed. Placentas, mice kidneys and uteri, fetal liver and brain were isolated rapidly after execution. Paraffin-embedded placentas were used for hematoxylin and eosin (H&E) staining, immunohistochemistry and fluorescence in situ hybridization. To assess the specificity of Siglec-6 placental expression, placental fluorescence in situ hybridization and IHC of the placenta, brain, liver, and uterus were conducted. Morphology and junctional zone/labyrinth architecture of placentas were assessed using H&E staining. IHC staining for CD31 (GB11063-1, Servicebio, Wuhan, China) was employed to evaluate the vasculature area of the placental labyrinth. Observation of placental mitochondrial morphology and structure was carried out using TEM, and measurement of mitochondrial area was performed using ImageJ. To analyze the kidney histologically, H&E staining and Periodic Acid–Schiff (PAS) colorations were performed. Glomerular integrity was assessed by a pathologist by expressing the percentage of glomerular capillary endothelial fenestrae as a percentage from 0 to 100%. Images of glomeruli were taken randomly using TEM.

### Statistical analysis

All data were expressed as the mean ± standard error of the mean (SEM). Student’s t-test was used to compare differences between two groups, and one-way ANOVA for three or more experimental groups. The GraphPad Prism 9 software was used for all statistical analyses. A value of *p* < 0.05 was considered to be statistically significant.

## Results

### Siglec-6 is upregulated in placental tissues of EO-PE

**Fig. 1:**
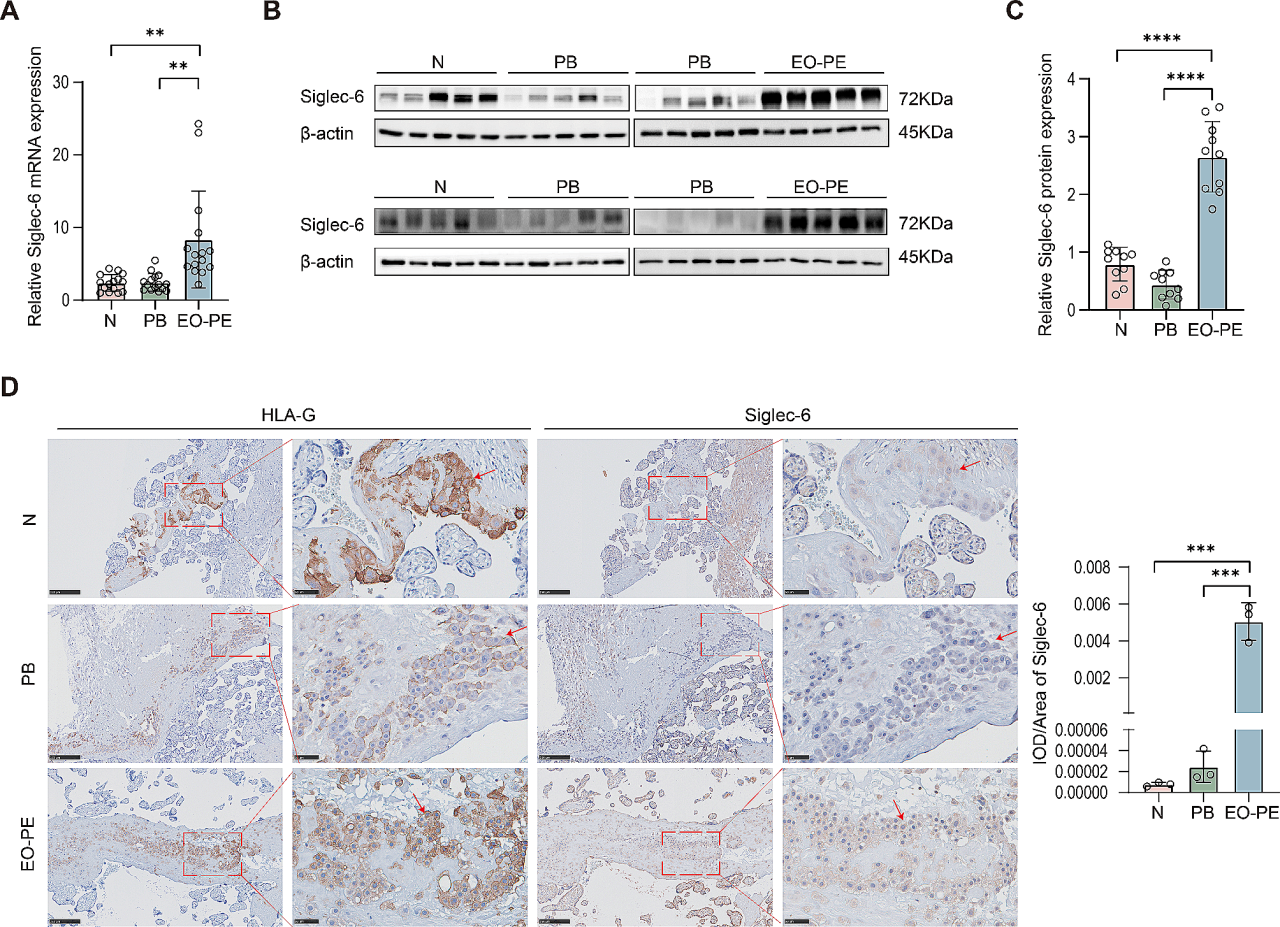
Aberrant expression of Siglec-6 is found in early-onset preeclamptic placenta. (**A**) qRT-PCR analysis of the Siglec-6 mRNA level in the placentas from full term pregnancies (*n* = 15), preterm birth (*n* = 15) and EO-PE (*n* = 15) patients. (**B**) Western blot analysis of Siglec-6 mRNA and protein levels in the placentas from full term pregnancies (*n* = 10), preterm birth (*n* = 10) and EO-PE (*n* = 10) patients. (**C**) Statistical analysis of protein densitometry quantification in Western blot (**B**). (**D**) Representative images of Siglec-6 expression and localization in the clinical samples (*n* = 3) by IHC analysis. Measurement of the IOD/area of immunohistochemical staining of Siglec-6. Red arrows point to EVTs (original magnification, 100×, scale bar = 250 μm; 400×, scale bar = 50 μm). N: normal pregnancy, PB: preterm birth, EO-PE: early-onset preeclampsia, HLA-G: human leucocyte antigen-G. All the statistical data were analyzed by Student’s t-test. All data are means ± SEM. **p<0.01, ***p<0.001, ****p<0.0001

Siglec-6 expression has been reported to be increased in placentas from preterm PE [17], but its localization and expression in the placenta has not been clearly defined. We analyzed the transcript and protein expression of Siglec-6 in placentas from full-term and preterm deliveries and from EO-PE patients by using real-time PCR and Western blotting. While the transcript and protein levels of Siglec-6 were comparable between full-term and preterm placentas, its transcript and protein expression levels were significantly increased in EO-PE placentas (Fig. 1A and C). In addition, we performed IHC to detect Siglec-6 localization in the placenta. HLA-G expression is restricted to EVTs and was therefore used to identify the EVT subpopulation. Siglec-6 was localized in the syncytiotrophoblasts and EVTs of placentas from all three groups. The IHC staining results showed that the staining intensity level of Siglec-6 was higher in EVTs from EO-PE placentas than in those from preterm and full-term placentas (Fig. 1C and D). Taken together, these data reveal that the expression of Siglec-6 is abnormal in EO-PE placentas.

### Siglec-6 regulates trophoblast cell migration and invasion

**Fig. 2:**
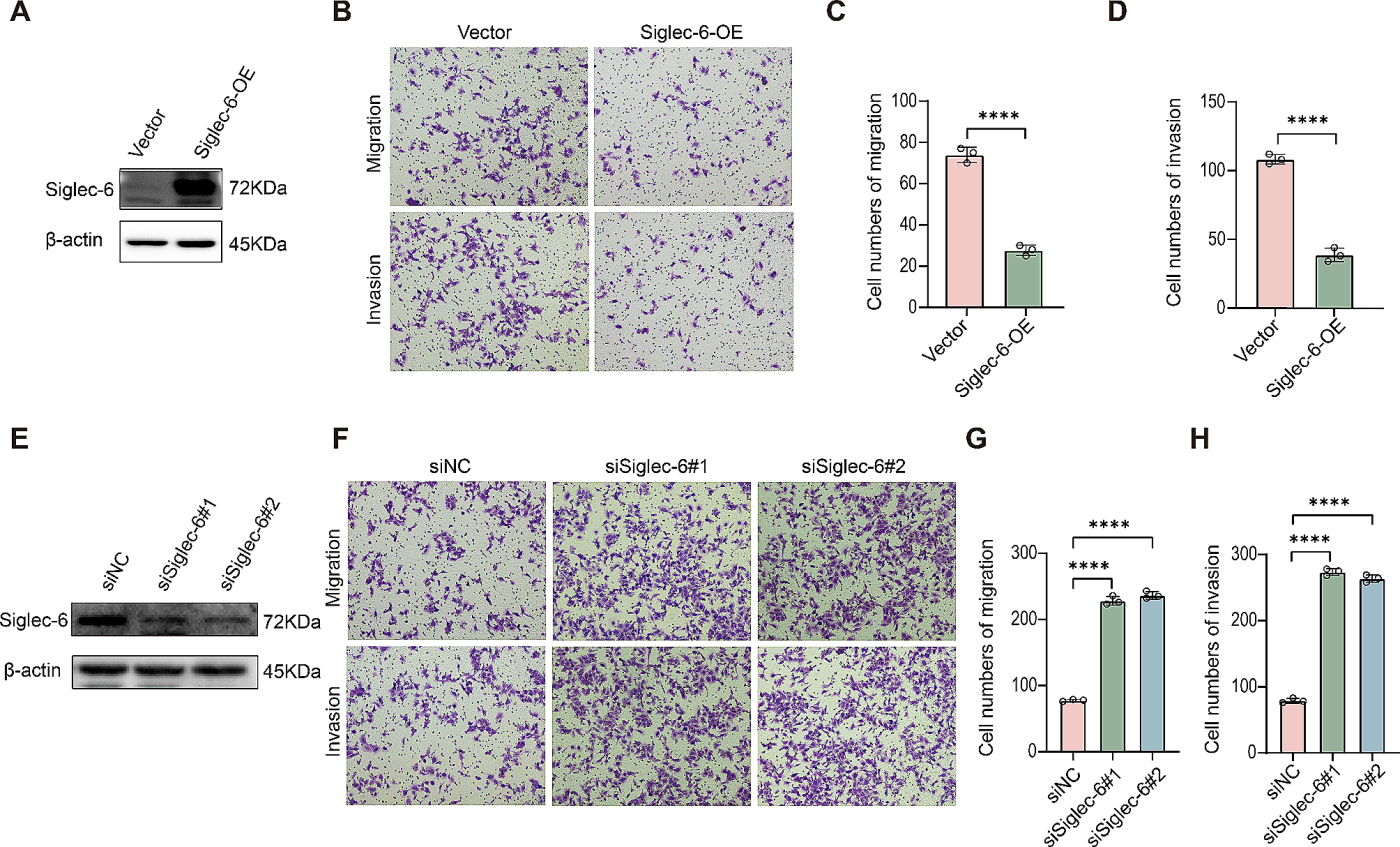
Siglec-6 is essential for trophoblast cell migration and invasion. (**A**) Western blot analysis of Siglec-6 protein levels in the stable HTR-8/SVneo cell line over-expressing Siglec-6. (**B**) Migration and invasion of stable HTR-8/SVneo cell line over-expressing Siglec-6 were determined by transwell assay. Representative images are shown. (**C**, **D**) The number of migrated and invaded HTR-8/SVneo cell line over-expressing Siglec-6 was counted. (**E**) Western blot analysis of the Siglec-6 protein level in HTR-8/SVneo cells transfected with siNC or siSiglec-6. (**F**) Migration and invasion of HTR-8/SVneo cells transfected with siNC or siSiglec-6 were determined by transwell assay. Representative images are shown. (**G**, **H**) The number of migrated and invaded HTR-8/SVneo cells transfected with siNC or siSiglec-6 was counted. All the statistical data were analyzed by Student’s t-test (two groups) or one-way ANOVA (above two groups). All data are means ± SEM of three independent experiments performed in triplicate. **p < 0.01; ****p < 0.0001

Based on the above observations, we speculated that increased Siglec-6 expression in EVTs is associated with PE. Although Siglec-6 has been reported to be involved in the regulation of trophoblast function, the findings are inconsistent [18–20], and there is also a lack of studies on the function of Siglec-6 in EVT. Therefore, we sought to investigate the role of Siglec-6 in HTR-8/SVneo and JAR cells. We stably overexpressed Siglec-6 in HTR-8/SVneo and JAR cells (Fig. 2A and Extended Data Fig. 1A and C). Then, we performed transwell assays to evaluate trophoblast cell migration and invasion and EdU assays to evaluate trophoblast cell proliferation. As shown in Fig. 2B and D and Extended Data Fig. 1E and G, Siglec-6 upregulation resulted in an apparent 60–80% inhibition of cell migration and invasion but had no effect on proliferation (Extended Data Fig. 2A and C). To further validate the important functions of Siglec-6 in trophoblast cells, we used two specific siRNAs to reduce Siglec-6 protein expression in HTR-8/SVneo and JAR cells. The effects of these siRNAs on Siglec-6 transcription and protein expression were verified by Western blotting (Fig. 2E and Extended Data Fig. 1D). Reduced Siglec-6 significantly promoted the migration and invasion of trophoblast cells (Fig. 2F and H and Extended Data Fig. 1H and J). These observations were compatible with the findings in the Siglec-6-overexpressing cell line, thereby further supporting the importance of Siglec-6 in trophoblast cells.

### Siglec-6 negatively regulates mitochondrial function in trophoblast cells

**Fig. 3:**
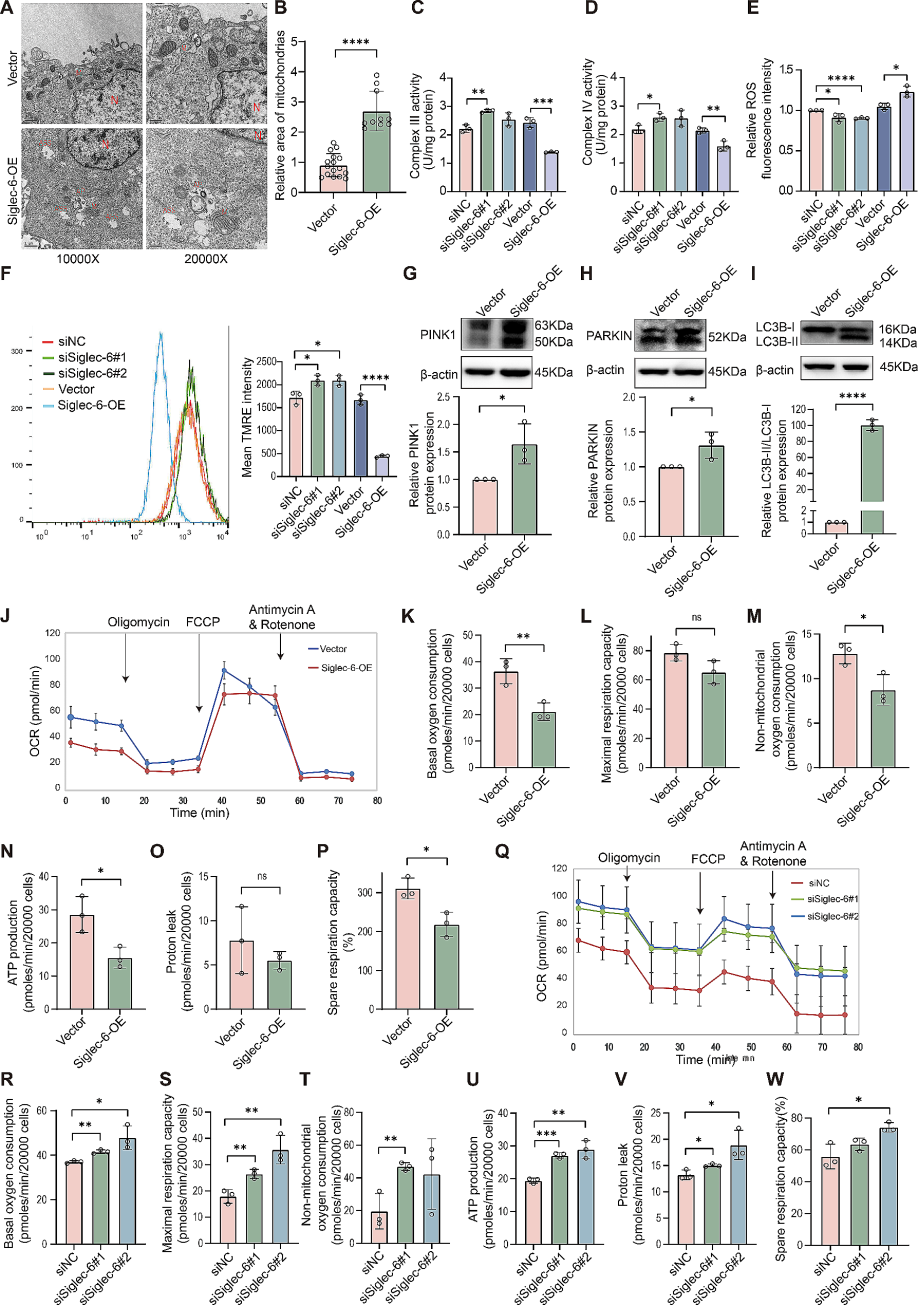
Effects of Siglec-6 on mitochondrial morphology and function in trophoblast cells. (**A**) TEM of the stable HTR-8/SVneo cell line over-expressing Siglec-6. N, nucleus; M, mitochondria; ASS, autophagy lysosome; AP, autophagic vesicle. (TEM, original magnification, 10,000×, scale bar = 1 μm; 20,000×, scale bar = 0.5 μm). (**B**) Mitochondrial surface area was measured. The activities of mitochondrial complex III (**C**) and IV (**D**) of the stable HTR-8/SVneo cell line over-expressing Siglec-6 and HTR-8/SVneo cells transfected with siNC or siSiglec-6 were measured. (**E**) ROS levels of the stable HTR-8/SVneo cell line over-expressing Siglec-6 and HTR-8/SVneo cells transfected with siNC or siSiglec-6 were measured. Flow cytometry was used to determine changes in mitochondrial membrane potential in in the stable HTR-8/SVneo cell line overexpressing Siglec-6, and in HTR-8/SVneo cells transfected with either siNC or siSiglec-6, using TMRE as the fluorescent probe. (**F**) Quantification of results shown in E. Western blot analysis of protein levels of PINK1 (**G**), PARKIN (**H**) or LC3B-II/ LC3B-I (**I**) in the stable HTR-8/SVneo cell line over-expressing Siglec-6, and quantified respectively. All protein levels are normalized to β-actin. (**J**, **Q**) Mitochondrial oxygen consumption rate (OCR) was recorded using Seahorse analyzer. Basal oxygen consumption (**K**, **R**), maximal respiration capacity (**L**, **S**), non-mitochondrial oxygen consumption (**M**, **T**), spare respiratory capacity (**N**, **U**), proton leak (**O**, **V**) and ATP production (**P**, **W**) were determined by using the Seahorse analyzer in the stable HTR-8/SVneo cell line over-expressing Siglec-6 and HTR-8/SVneo cells transfected with siNC or siSiglec-6. Results are shown as mean ± SEM. All the statistical data were analyzed by Student’s t-test (**B**, **G**-**I**, **K**-**P**) or one-way ANOVA (**C**-**F**, **R**-**W**). *p<0.05, **p<0.01, ***p<0.001, ****p<0.0001

Mitochondrial dysfunction has been implicated in various cell processes, including proliferation, differentiation, and migration/invasion. Previous studies have suggested that members of the Siglec family are involved in regulating mitochondrial function. Therefore, we speculated that Siglec-6 participates in the migration and invasion of trophoblast cells by regulating mitochondrial function. To test this hypothesis, we first determined the effect of Siglec-6 overexpression on trophoblast mitochondrial structure by TEM. As shown in Fig. 3A and B and Extended Data Fig. 4A and B, overexpression of Siglec-6 resulted in a series of pathological changes in trophoblast cell mitochondria, such as swelling, reduced number of mitochondrial cristae, irregular arrangement, and localized thinning and lightening of the mitochondrial matrix, compared with the control cells, which was consistent with the mitochondrial characteristics in the EO-PE placenta. In addition, multiple autophagic lysosomes and autophagic vesicles were visible in Siglec-6-overexpressing trophoblast cells. Because abnormal mitochondrial morphology is often linked to altered energy metabolism, we examined the mitochondrial respiratory chain complex activity in HTR-8/SVneo cells. The results showed that the activities of mitochondrial complex III and complex IV were reduced by 30–50% in Siglec-6-overexpressing trophoblast cells, while Siglec-6 knockdown resulted in a sustained 20–30% increase in the activities of complex III and complex IV (Fig. 3C and D). Since Siglec-6 is involved in regulating the activity of mitochondrial respiratory chain complex III and complex IV, which together form one of the main sources of ROS, we investigated the effect of Siglec-6 on ROS generation in trophoblast cells. The results showed that overexpression of Siglec-6 enhanced ROS generation in HTR-8/SVneo cells, whereas knockdown of Siglec-6 inhibited ROS generation (Fig. 3E), suggesting that Siglec-6 promotes trophoblast ROS generation. We also analyzed mitochondrial membrane potential (MMP), the stabilization of which is a prerequisite for mitochondrial function [21]. We assessed the MMP in HTR-8/SVneo cells by TMRE, as the fluorescence intensity of TMRE is proportional to the MMP. We found that the average fluorescence intensity of TMRE was significantly decreased in cells overexpressing Siglec-6, whereas the average fluorescence intensity of TMRE was elevated in Siglec-6 knockdown trophoblast cells (Fig. 3F), indicating that Siglec-6 decreased the MMP of HTR-8/SVneo cells. It has been reported that excessive ROS accumulation induces mitochondrial oxidative stress damage, leading to an increase in mitochondrial autophagy [22]; in addition, a decrease in membrane potential induces an increase in PINK1- and Parkin-mediated mitochondrial autophagy. Considering the impact of Siglec-6 on ROS generation and mitochondrial membrane potential, we investigated its influence on the expression levels of mitochondrial autophagy-related proteins in HTR-8/SVneo cells. Western blot results showed that overexpression of Siglec-6 in HTR-8/SVneo cells significantly increased the expression of PINK1 and PARKIN and the ratio of LC3B-II/LC3B-I compared with those in the control group (Fig. 3G and I), indicating that Siglec-6 promotes mitochondrial autophagy in trophoblast cells. To characterize real-time mitochondrial respiration in trophoblast cells, we used a Seahorse extracellular flux analyzer to determine the OCRs of the cells. Siglec-6 overexpression resulted in significantly lower mitochondrial function parameters in HTR-8/SVneo and JAR cells (Fig. 3J and P and Extended Data Fig. 4C and I), including OCR, basal oxygen consumption, maximal respiration capacity, nonmitochondrial oxygen consumption, ATP production, proton leak and spare respiratory capacity. Conversely, HTR-8/SVneo and JAR cells with Siglec-6 knockdown showed elevated mitochondrial function parameters compared with controls (Fig. 3Q and W and Extended Data Fig. 4J and P). Taken together, these results reveal that Siglec-6 is essential for trophoblast mitochondrial morphology and function.

**Fig. 4:**
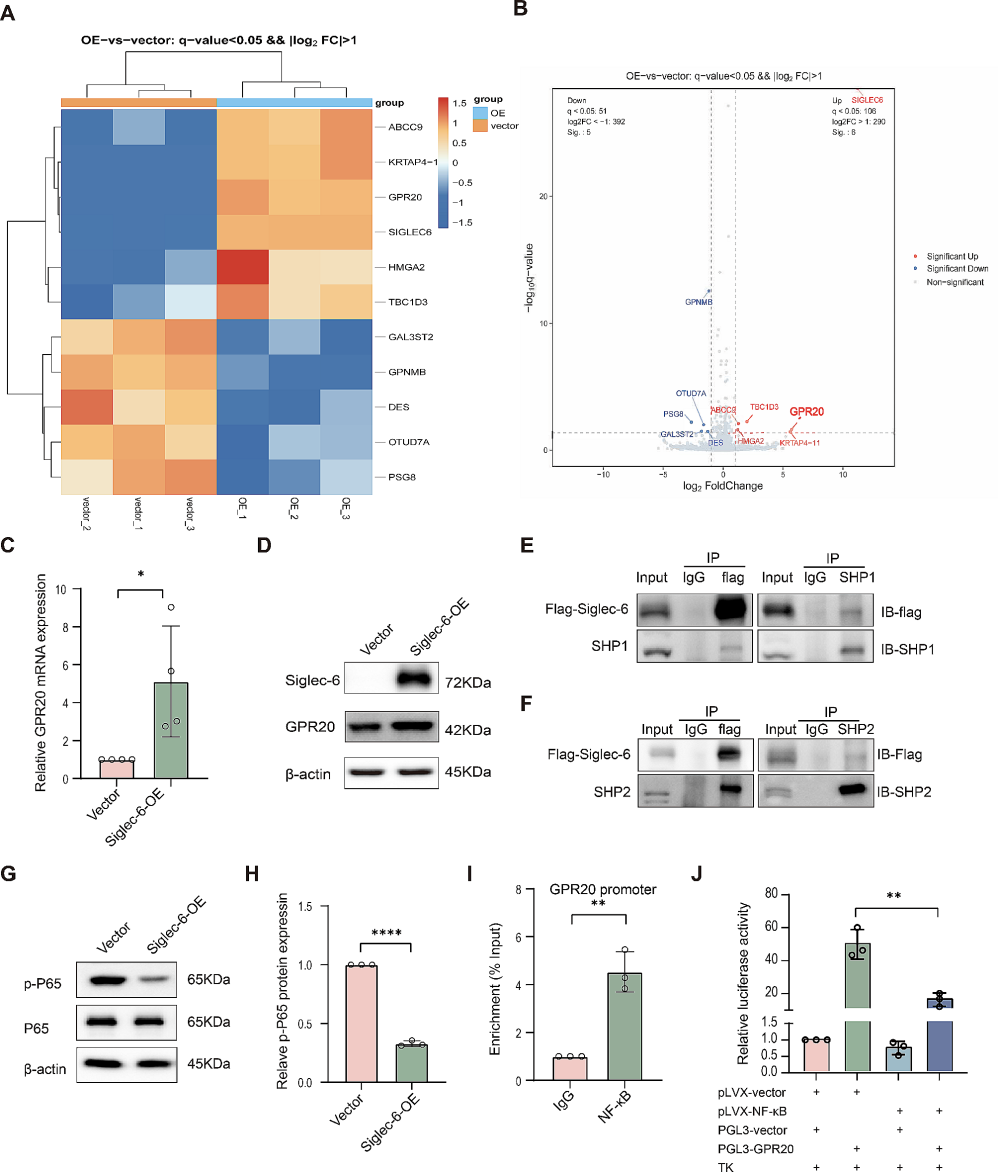
Siglec-6 upregulates GPR20 expression through SHP1/SHP2-NF-κB pathway in trophoblast cells. Heatmap (**A**) and volcano plot (**B**) of differentially expressed genes between the vector and the Siglec-6 overexpression group. (**C**) qRT-PCR analysis of the GPR20 mRNA level in the stable HTR-8/SVneo cell line over-expressing Siglec-6. Data are the mean ± SEM (*n* = 3). Statistical data were analyzed by Student’s t-test. *p < 0.05. (**D**) Western blot analysis of Siglec-6 and GPR20 protein levels in the stable HTR-8/SVneo cell line over-expressing Siglec-6. Stable HTR-8/SVneo cell lysis was incubated with anti-Flag or anti-SHP1/SHP2, then analyzed by western blot using specific antibody to SHP1 (**E**)/SHP2 (**F**) or Flag. (**G**) Western blot analysis of protein levels of pSer536-p65 or p65 in the stable HTR-8/SVneo cell line over-expressing Siglec-6. (**H**) Quantification of the expression of the pSer536-p65 protein in Western blot G. Data are the mean ± SEM (*n* = 3). Statistical data were analyzed by Student’s t-test. ****p < 0.0001. (**I**) The GPR20 promoter was detected with ChIP assays with anti-NF-κB/p65 antibody, and then analyzed by quantitative PCR. The value represents the effect of the NF-κB/p65 occupancies at the promoter of GPR20. Data are the mean ± SEM (*n* = 3). Statistical data were analyzed by Student’s t-test. **p < 0.01. (**J**) 293T cells were transfected with NF-κB/p65 overexpression plasmid or vector control for 48 h, after which the transfected cells were co-transfected with a wild-type GPR20-luciferase transcriptional reporter. Data are expressed as mean fold induction ± SEM of luciferase activity relative to controls (*n* = 3). Statistical data were analyzed by two-way ANOVA. **p < 0.01

### Siglec-6 upregulates GPR20 expression through the SHP1/SHP2-NF-κB pathway in trophoblast cells

We subsequently carried out an RNA-sequencing analysis on HTR-8/SVneo cells overexpressing Siglec-6 to explore the molecular mechanisms underlying the impact of Siglec-6 on trophoblast cell mitochondrial structure and function. We identified a total of 11 differentially expressed genes using systematic clustering analysis with a significance threshold of q-value < 0.05 and |log2FC|>1.0. Of those, 5 genes were downregulated, and 6 genes were upregulated. Notably, GPR20 showed the highest upregulation among all factors other than Siglec-6 (Fig. 4A and B). The RNA-sequencing results were confirmed by qRT‒PCR and Western blot, which showed an increase in GPR20 mRNA and protein levels in HTR-8SVneo cells overexpressing Siglec-6, consistent with the RNA sequencing results (Fig. 4C and D). Several members of the Siglec family have demonstrated the ability to prevent downstream NF-κB activation and initiate inhibitory signaling by recruiting SHP1/SHP2 proteins through the intracellular immunoreceptor tyrosine-based inhibitory motif (ITIM) structural domain [23]. Additionally, Siglec-6 contains both an ITIM and an ITIM-like motif in its cytoplasmic tail [24]. Our analysis, performed using the JASPAR database tool (http://jaspar.genereg.net/), identified a nuclear factor NF-κB binding site in the GPR20 promoter with the potential to regulate GPR20 expression. Therefore, we hypothesized that Siglec-6 may regulate GPR20 expression via the SHP1/SHP2-NF-κB signaling pathway. To test this hypothesis, we performed coimmunoprecipitation assays to determine whether there is an interaction between Siglec-6 and SHP1 or SHP2. Initially, HTR-8/SVneo cells were transfected with Flag-tagged Siglec-6 plasmid. Then, the lysates were immunoblotted and probed with anti-Flag, anti-SHP1, and anti-SHP2 antibodies. As shown in Fig. 4E and F, Siglec-6 interacted with SHP1 and SHP2 in trophoblast cells. Furthermore, we detected the level of NF-κB phosphorylated at serine 536 in HTR-8/SVneo cells through Western blotting. The results indicated that the NF-κB phosphorylation level was significantly lowered in HTR-8/SVneo cells overexpressing Siglec-6 (Fig. 4G and H). To further clarify the mechanism by which NF-κB regulates GPR20 expression, we carried out a ChIP experiment to assess the enrichment of NF-κB at the GPR20 promoter, which showed that NF-κB localized to the GPR20 promoter (Fig. 4I). Moreover, luciferase activity was significantly downregulated in 293T cells when the GPR20 plasmid was cotransfected with the NF-κB overexpression plasmid (Fig. 4J). This suggested that NF-κB repressed the transcriptional activity of GPR20. Collectively, these findings indicated that Siglec-6 inhibited the phosphorylation of NF-κB by recruiting SHP1/2, which in turn reduced the inhibitory effect of NF-κB on the transcriptional activity of GPR20, resulting in an increase in GPR20 expression.

### GPR20 is a key gene downstream of Siglec-6 that regulates the morphology and function of trophoblast mitochondria

**Fig. 5:**
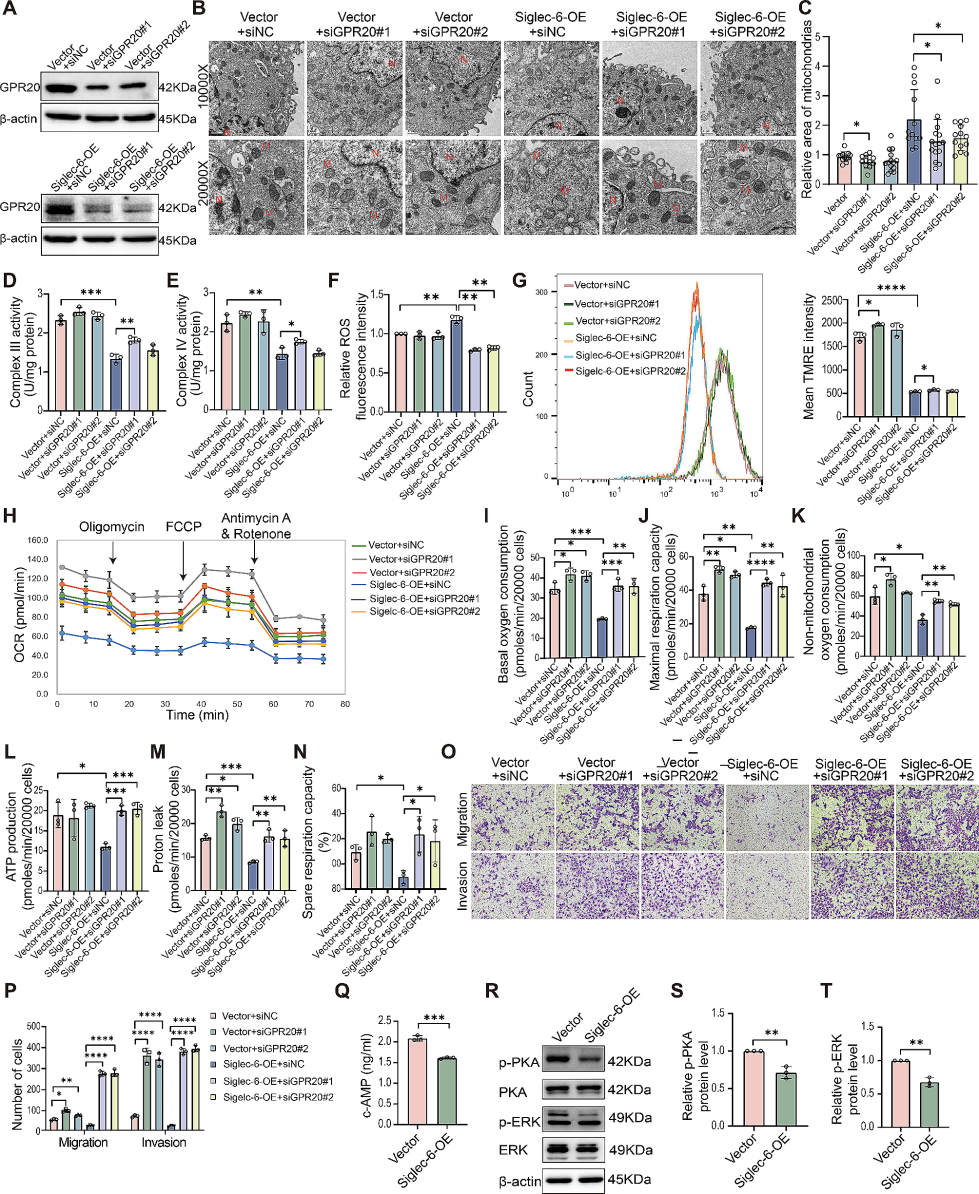
GPR20 acts as a key factor downstream of Siglec-6 to regulate the function of mitochondria in trophoblast cells. (**A**) Western blot analysis of GPR20 protein levels in HTR-8/SVneo cells transfected with siNC or siGPR20. (**B**) TEM of the stable cell lines. N, nucleus. M, mitochondria. (**C**) Mitochondrial surface area was measured. The activities of mitochondrial complex III (**D**) and IV (**E**) of the stable cell lines were measured. (**F**) ROS levels of the stable cell lines were measured. (**G**) Flow cytometric determination of mitochondrial membrane potential changes in the stable cell lines by TMRE. (**H**) Mitochondrial OCR was recorded using Seahorse analyzer. Basal oxygen consumption (**I**), maximal respiration capacity (**J**), non-mitochondrial oxygen consumption (**K**), ATP production (**L**), proton leak (**M**), and spare respiratory capacity (**N**) were determined by using the Seahorse analyzer in the stable cell lines. (**O**) Migration and invasion of the stable cell lines were determined by transwell assay. (**P**) The number of migrated and invaded cells was counted. (**Q**) c-AMP levels of the stable cell lines were measured. (**R**-**T**) Western blot analysis of protein levels of pThr197-PKA, PKA, pThr202/Tyr204-ERK or ERK and quantification of the expression of the pThr197-PKA or pThr202/Tyr204-ERK protein in the stable HTR-8/SVneo cell line over-expressing Siglec-6. Data are the mean ± SEM (*n* = 3). All the statistical data were analyzed by Student’s t-test (two groups) or two-way ANOVA (above two groups). *p < 0.05, **p < 0.01, ***p < 0.001, ****p < 0.0001

According to previous reports, GPR20 has an inhibitory effect on the c-AMP signaling pathway, which plays a critical role in regulating mitochondrial function [25]. Based on this observation, we speculated that GPR20 acts as a significant downstream gene of Siglec-6 in regulating trophoblast cell mitochondrial function. To test this hypothesis, we performed rescue experiments in trophoblast cells. For this purpose, we transfected Siglec-6-overexpressing trophoblast cells with siNC or siGPR20 (Fig. 5A). We found that knockdown of GPR20 was able to moderately improve the Siglec-6 overexpression-induced morphopathological changes in mitochondria (Fig. 5B and C). Knockdown of GPR20 partially restored the activity of mitochondrial complexes III and IV in HTR-8/SVneo cells, which were reduced due to Siglec-6 overexpression (Fig. 5D and E). Furthermore, it partially reinstated the increased production of ROS (Fig. 5F) and the decreased mitochondrial membrane potential (Fig. 5G) and OCR (Fig. 5H and N) in HTR-8/SVneo cells. It was also discovered that knocking down GPR20 in HTR-8/SVneo cells overexpressing Siglec-6 led to increased trophoblast migration and invasion, as shown in Fig. 5O and P. Then, we performed ELISAs to determine the c-AMP levels; the results showed that the concentration of c-AMP was reduced in HTR-8/SVneo cells overexpressing Siglec-6 (Fig. 5Q). Western blot results revealed that the levels of PKA phosphorylated at threonine 197 and ERK phosphorylated at threonine 202/tyrosine 204 were decreased, as shown in Fig. 5R and T, suggesting that Siglec-6 inhibits the downstream c-AMP/PKA/ERK signaling pathway by promoting the expression of GPR20. The results above demonstrate that GPR20 can rescue the functional deficiencies caused by Siglec-6 overexpression in terms of aberrant mitochondrial morphology and function, as well as reduced cell migration and invasion. Altogether, these findings indicate that GPR20 is a crucial downstream gene in the regulation of mitochondrial morphology and function in trophoblast cells by Siglec-6.

### GPR20 and NF-κB are aberrantly expressed in EVTs from EO-PE

**Fig. 6:**
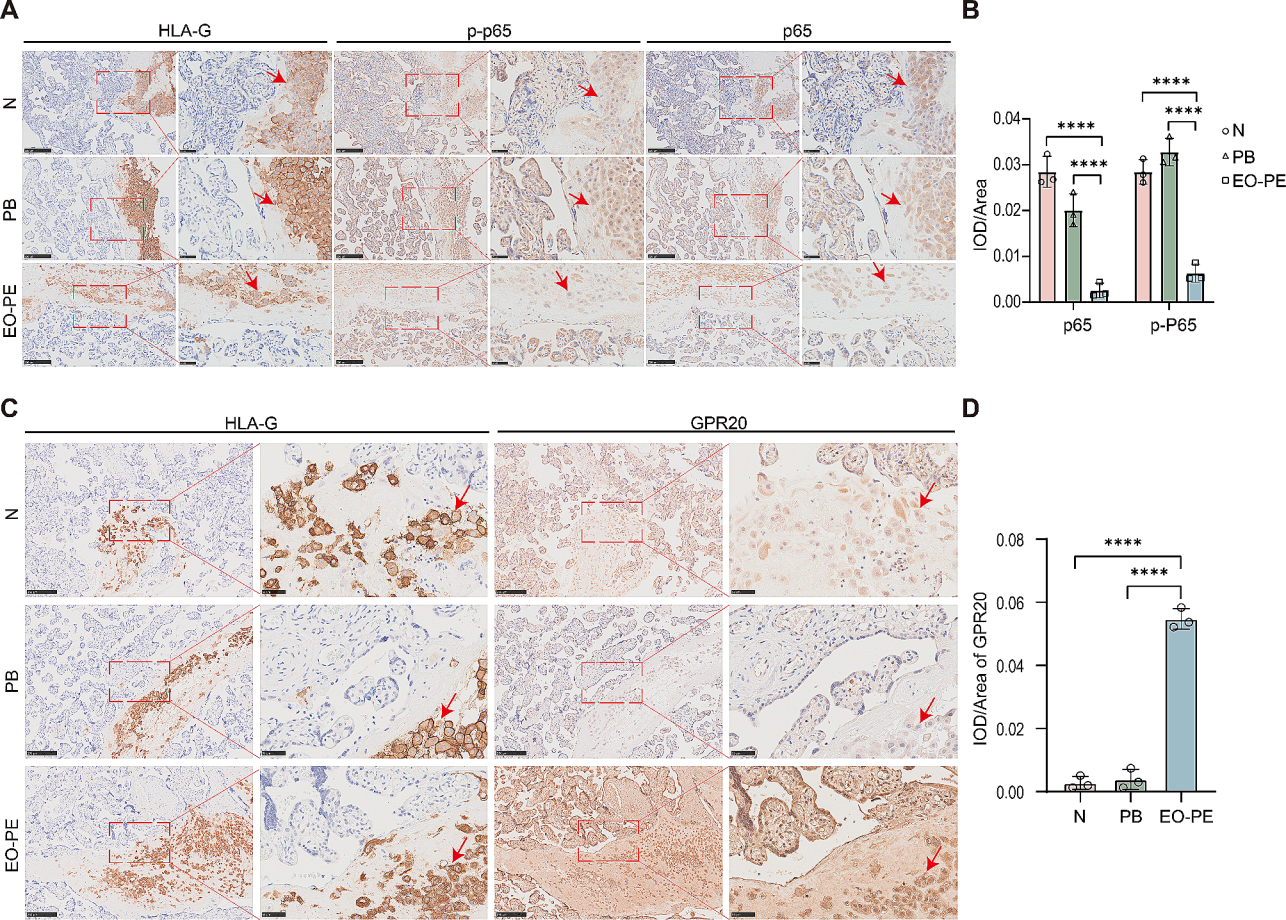
GPR20 and NF-κB are aberrantly expressed in decidua-embedded EVTs from EO-PE. (**A**) Representative images of HLA-G, pSer536-p65 or p65 expression and localization in the placentas from full term pregnancies, preterm birth and EO-PE patients by IHC analysis (*n* = 3). (**B**) Measurement of the IOD/area of immunohistochemical staining of pSer536-p65 or p65. (**C**) Representative images of HLA-G or GPR20 expression and localization in the clinical samples. (**D**) Measurement of the IOD/area of immunohistochemical staining of GPR20. Red arrows point to EVTs (original magnification, 100×, scale bar = 250 μm; 400×, scale bar = 50 μm). N: normal pregnancy, PB: preterm labor, EO-PE: early onset preeclampsia, HLA-G: human leucocyte antigen. Results are shown as mean ± SEM. All the statistical data were analyzed by Student’s t-test (**D**) or two-way ANOVA (B). ****p<0.0001

To further evaluate the possible cause of reduced trophoblast motility in EO-PE, we used IHC to investigate GPR20, p65 phosphorylated at serine 536 and total p65 expression in EVTs from full-term and preterm deliveries and from EO-PE patients. The expression of HLA-G was used to identify EVTs. The IHC staining results indicated that the staining intensities of phosphorylated p65 and p65 were lower in EVTs from EO-PE than in those from full-term and preterm deliveries (Fig. 6A and B), while the staining intensity level of GPR20 was higher in EVTs from EO-PE than in those from full-term and preterm deliveries (Fig. 6C and D). In summary, these results reveal that GPR20 and NF-κB signaling are dysregulated in EO-PE.

### Overexpression of Siglec-6 in the placenta specifically leads to a PE-like phenotype in pregnant mice

**Fig. 7:**
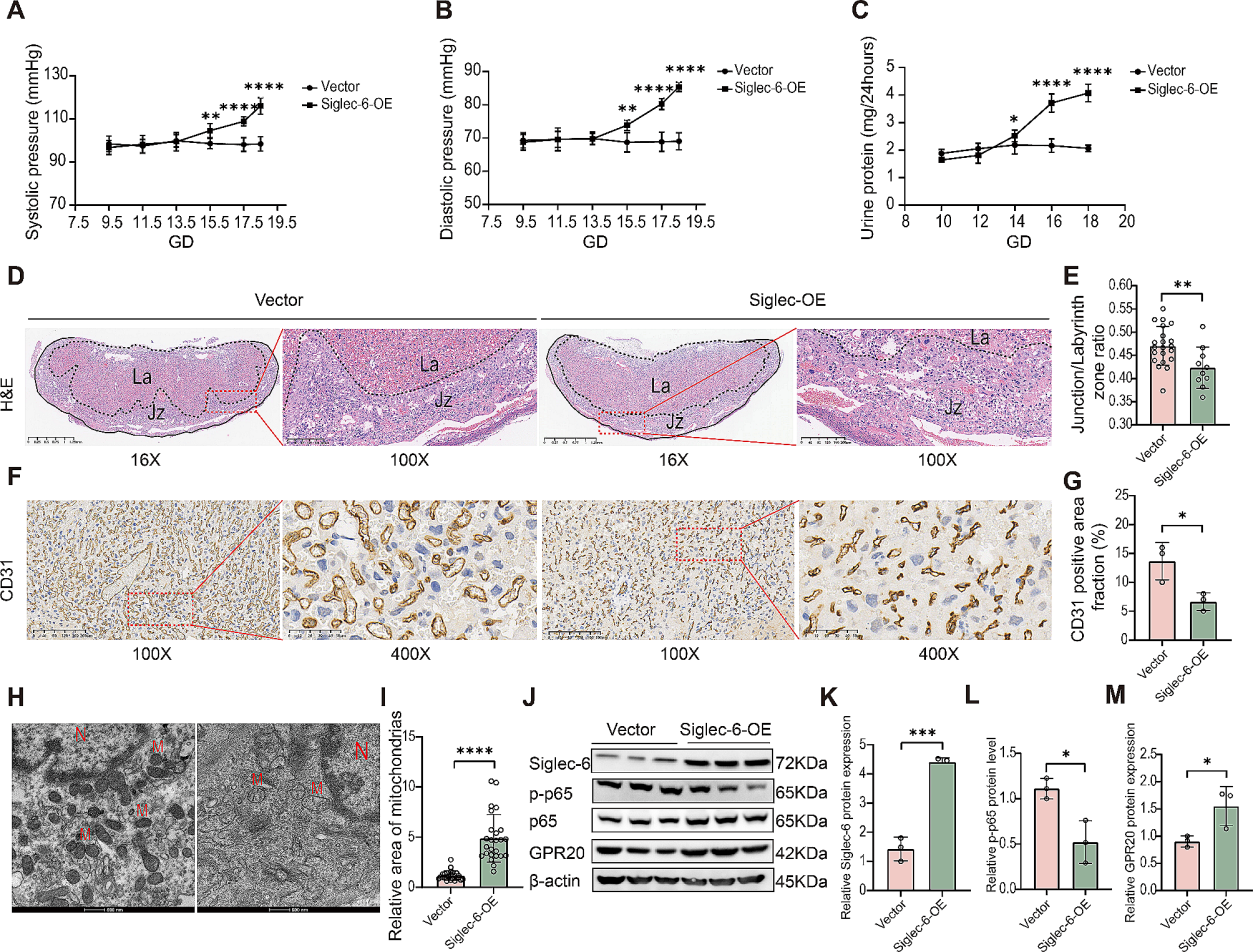
Overexpression of Siglec-6 in the placenta specifically leads to a PE-like phenotype in pregnant mice. The SBP (**A**), DBP (**B**), total urinary protein (**C**) of pregnant mice. (**D**) **H**&**E** staining of mouse placentas. JZ, junctional zone; L, labyrinth. (**E**) Ratio of junctional zone area to labyrinth zone area in mice. (**F**) IHC staining for CD31 in mouse placental tissues. Brown color indicates positive staining for CD31. (**G**) The area fraction of CD31 staining in labyrinth was measured. (**H**) TEM of mouse placentas. N, nucleus; M, mitochondria. Scale bar: 500 nm. (**I**) Mitochondrial surface area was measured. (**J**-**M**) Western blot analysis of protein levels of pSer536-p65, p65 or GPR20 and quantification of pSer536-p65 or GPR20 protein expression in mouse placentas. Results are shown as mean ± SEM. All the statistical data were analyzed by Student’s t-test (**E**, **G**, **I**, **K**-**M**) or one-way ANOVA (**A**-**C**). *p<0.05, **p<0.01, ***p<0.001, ****p<0.0001

Since abnormal placentation is the focus of EO-PE pathogenesis and Siglec-6 is specifically expressed in the human placenta, we observed the pregnancy outcomes of mice overexpressing Siglec-6 specifically in the mouse placenta. To conduct this experiment, we utilized lentivirus to infect only the trophectoderm during the blastocyst stage and generated a pregnant mouse model that overexpresses Siglec-6 specifically in the placenta [26] (Extended Data Fig. 5A and C). We then assessed hypertension and proteinuria, which are typical characteristics of PE, in the pregnant mouse model. As shown in Fig. 7A and B, compared with controls, Siglec-6-overexpressing pregnant mice showed increases in both systolic and diastolic blood pressure beginning at GD15.5 and an increase in total 24-hour urine protein beginning at GD16, all of which continued until the end of pregnancy (Fig. 7A and C). Pregnant mice were euthanized at GD18.5, and the weights of the placentas and fetuses were recorded. No significant differences in the weights were found between Siglec-6-overexpressing and control mice (Extended Data Fig. 6C and E). Because blood pressure in patients with PE rapidly returns to normal after delivery, we also examined blood pressure after delivery in the pregnant mouse model; we observed a parallel phenomenon in Siglec-6-overexpressing mice (Extended Data Fig. 6A and B). Then, the effects of Siglec-6 on placental morphology were determined by H&E staining, and the results showed that overexpression of Siglec-6 resulted in abnormalities in the placental structure of the pregnant mice, as indicated by a reduction in the ratio of the junctional zone to the labyrinthine zone (Fig. 7D and E). Furthermore, IHC analysis of the placental vasculature with CD31 staining showed that overexpression of Siglec-6 led to a reduced vessel count and lumen diameter in the placenta, suggesting impaired maternal–fetal exchange (Fig. 7F and G). PE is typically associated with renal lesions such as glomerular capillary endothelial proliferation and podocyte injury. Therefore, we carried out IHC and TEM analysis to detect the effects of Siglec-6 overexpression on the kidneys of pregnant mice. The histologic analysis revealed that the maternal kidneys of Siglec-6-OE mice had a normal structure and that the glomerular area was similar to that in the control group (Extended Data Fig. 6F). Nevertheless, the TEM results demonstrated glomerular endothelial hyperplasia and incomplete endothelial interstitial space (Extended Data Fig. 6G), indicating that the glomerular ultrastructure was damaged in the Siglec-6-overexpressing mice. Furthermore, we inspected the placental ultrastructure of Siglec-6-OE mice by TEM, which revealed pathological changes such as trophoblast mitochondrial swelling and the reduction or disappearance of mitochondrial cristae (Fig. 5H). Finally, we detected the protein levels of Siglec-6, NF-κB, and GPR20 in the mouse placenta, which revealed reduced phosphorylated NF-κB and elevated GPR20 levels in the Siglec-6-overexpressing mice (Fig. 7I and K). Taken together, these findings reveal that overexpression of Siglec-6 in the placenta may result in PE-like adverse reproductive outcomes through the NF-κB/GPR20 pathway.

## Discussion

Preeclampsia is a condition unique to humans that has no effective predictive, preventive or therapeutic approaches due to its unclear etiology [10]. Compared with late-onset preeclampsia, early-onset preeclampsia is associated with significant deficiencies in uterine spiral artery remodeling, resulting in inadequate placental perfusion, hypoxia, and reperfusion injuries in EO-PE [3]. In contrast, late-onset preeclampsia is frequently associated with maternal endothelial cell dysfunction and is believed to be impacted by preexisting maternal disorders that may affect endothelial integrity [27]. Numerous studies have demonstrated a significant increase in Siglec-6 expression in preeclamptic placentas compared with placentas from normal pregnancies [28, 29]. Although it has been demonstrated that Siglec-6 overexpression affects trophoblast proliferation, apoptosis, migration, and invasion [30, 31], the contribution of Siglec-6 to the development of EO-PE and the underlying molecular mechanisms remain unclear.

According to a report by Xiaonian Guan et al., the development of EO-PE is mediated by Siglec-6, which impairs vascular endothelial cell function through downregulation of Wnt6/β-Catenin signaling and inhibition of Wnt6 secretion by a paracrine mechanism [12]. The study demonstrated that Siglec-6 was predominantly expressed in syncytiotrophoblasts and EVTs. Moreover, the expression of Siglec-6 was found to be significantly high in EVTs of the placenta from EO-PE, which is consistent with the findings of our study. However, it is worth noting that Guan’s study concluded that overexpression of Siglec-6 did not substantially affect the proliferation, migration, or invasion of trophoblast HTR-8/SVneo cells. In contrast, our study found that overexpression of Siglec-6 significantly hampered the ability of trophoblasts to migrate and invade. To verify this conclusion, we also analyzed the impact of Siglec-6 overexpression on trophoblast JAR cells and obtained the same results as in HTR-8/SVneo cells. The inconsistent conclusions of the two studies may be attributed to differences in the level of overexpression of Siglec-6 between Guan et al. and our study. Specifically, in their study, Siglec-6 transcript levels were elevated by 200,000-fold and protein levels by 10-fold compared with the control, while in our study, Siglec-6 transcript levels were elevated by 15,000-fold and protein levels were elevated 5 ~ 6-fold compared to the control. It is possible that the excessive upregulation of Siglec-6, as observed in Guan et al., does not fully reflect its actual function. Furthermore, we created two small interfering RNAs targeting Siglec-6 and revealed that the downregulation of Siglec-6 could enhance the migration and invasion of trophoblast cells in transfected trophoblast HTR-8/SVneo and JAR cells. In conclusion, we have demonstrated the regulatory effects of Siglec-6 on trophoblast cell migration and invasion via both overexpression and knockdown techniques.

Leptin is the only known nonglycosylated natural ligand of Siglec-6. It binds to Siglec-6 and moderates the migration and invasion of choriocarcinoma BeWo cells [30]. We therefore also examined the influence of leptin in controlling the migration and invasion of EVTs (Extended Data Fig. 3). The results indicated that leptin could enhance the migration and invasion of HTR-8/SVneo cells in a concentration-dependent manner. While knocking down Siglec-6 did not impact the effects of leptin on trophoblast migration and invasion, knocking down leptin R blocked the changes in the migration and invasion ability of the cells with increasing leptin concentrations. Thus, we suggest that leptin mainly regulates the migration and invasion of trophoblast cells through leptin R rather than Siglec-6. The above results suggest that the suppression of trophoblast cell migration and invasion by Siglec-6 is independent of leptin, and there may be other ligands that are involved in regulating trophoblast cell biological functions through Siglec-6; this point requires further investigation.

PE is closely associated with trophoblast mitochondrial dysfunction [32, 33], and the results of this study are in accordance with previous findings that have reported abnormalities in placental trophoblast mitochondrial morphology in severe PE [8]. Furthermore, anomalous expression and activity of the respiratory chain complex have been detected in the mitochondria of placental trophoblasts in PE [34]. Mitochondrial respiratory chain complexes III and IV play a fundamental role in ROS generation [35], and reductions in their activity result in increased ROS production. The present study has demonstrated that Siglec-6 exerts a regulating influence upon the activity of mitochondrial respiratory chain complexes III and IV. Conversely, no effect was observed upon the activity of complexes I and II (Extended Data Fig. 7). In addition, overexpression of Siglec-6 significantly inhibited basal oxygen consumption, ATP production and spare respiration capacity (SRC) of trophoblast cells. It can be posited that SRC is a measure of mitochondrial fitness, which is indicative of the functionality of ‘healthy’ mitochondria [36]. This is contingent upon the activity of respiratory chain components. It has been demonstrated that the activity levels of mitochondrial complexes I and II exert an influence on SRC [37]. Furthermore, in myeloid leukaemia cells, a reduction in the activity of mitochondrial complex III has been postulated as a potential causal factor for the observed decrease in SRC levels [38]. Additionally, it has been shown that oxidative stress conditions result in a decline in the enzymatic activity of mitochondrial complex IV, which in turn contributes to a reduction in SRC levels [39]. Thus, the SRC is low in the Siglec-6-OE group considering that the defected complex III and IV activity.

It is noteworthy that the non-mitochondrial oxygen consumption of trophoblast cells was also negatively regulated by Siglec-6, although with a relatively weak effect. This suggests that Siglec-6 may be involved in the cellular non-mitochondrial oxygen consumption. It has been demonstrated that in dendritic cells, within the lipid microdomain, Siglec-E can exert a negative regulatory effect on LOX-1-mediated immune activation [40]. LOX-1 is a member of the dioxygenase family, which incorporates molecular oxygen into polyunsaturated fatty acids to form fatty acid hydroperoxides [41]. This indicates that Siglec-E may regulate cellular non-mitochondrial oxygen consumption. Nevertheless, further investigation is required to determine whether Siglec-6 plays a role in non-mitochondrial oxygen consumption in trophoblast cells and to gain insight into the underlying regulatory mechanisms.

To further investigate the molecular mechanisms through which Siglec-6 regulates mitochondrial function, we carried out transcriptome sequencing of HTR-8/SVneo cells overexpressing Siglec-6. We focused on GPR20, which, with the exception of Siglec-6, exhibited the greatest increase in expression levels. The intracellular ITIM structural domain in most Siglec family members can recruit SHP1/SHP2 proteins, which subsequently inhibit the activation of the downstream transcription factor NF-κB and initiate an inhibitory signaling effect [23]. According to Stefanski and colleagues’ report, Siglec-6 recruits SHP2 through tyrosine phosphorylation of Src kinase, but these authors were unable to detect the presence of SHP1 in HTR-8/SVneo cells [42]. However, our results revealed that SHP1 is expressed in HTR-8/SVneo cells and interacts with Siglec-6. One possible reason for the nondetection of SHP1 in HTR-8/SVneo cells by Stefanski et al. could be their choice of Jurkat cells as a positive control. Based on the analysis of SHP1 expression levels in various human tissues [23], SHP1 expression is significantly higher in lymph nodes than in the placenta. Hence, the SHP1 expression in HTR-8/SVneo cells may have been masked by its expression in Jurkat cells during the Western blot experiments. Additionally, the use of SHP1 antibodies with different potencies in the two experiments may have affected the outcomes. We have demonstrated that the overexpression of Siglec-6 suppresses the phosphorylation of NF-κB, a protein that represses the transcription of GPR20 by binding as a transcriptional suppressor.

GPR20 is a class-A orphan G protein-coupled receptor that activates Gi proteins in the absence of any known ligand [25]. It has also been suggested to play an important role in mitochondrial function [25]. According to Fang et al., GPR20 caused the downregulation of c-AMP, resulting in a decrease in the c-AMP/PKA/ERK signaling pathway, which affected cellular mitochondrial function. In line with this finding, we found that GPR20 can rescue the functional deficiencies caused by Siglec-6 overexpression in aberrant mitochondrial morphology and function through the c-AMP/PKA/ERK signaling pathway. It is worth noting that the GPCR family is one of the largest gene families present in the human genome, and it is also a critical target for drug development [43]. Dysregulation of the GPCR expression profile has been implicated as a potential factor in the development of PE. Several target GPCRs, along with their agonists or antagonists, have been proven to have substantial clinical potential in preventing PE and extending gestation [44, 45]. Our study indicated that GPR20 expression is upregulated in the placental EVTs of PE, suggesting GPR20 as a potential novel target for the prevention or treatment of this disease.

Taking advantage of the ability of lentiviruses to exclusively infect the trophectoderm at the blastocyst stage, we established a pregnant mouse model that overexpresses Siglec-6 specifically in the placenta to study the critical role of Siglec-6 in the development of preeclampsia in vivo. This model mouse placenta presented a reduction in the area of the junctional zone and a smaller vascular lumen in the labyrinthine area. Although H&E staining indicated no apparent abnormalities in the kidneys of pregnant mice, TEM demonstrated glomerular endothelial hyperplasia and incomplete endothelial gaps, implying that the kidneys of pregnant mice were also pathologically altered. Notably, our data are partially inconsistent with the preceding result that the placental and fetal weights are decreased in the PE model. We speculate that this discrepancy is likely due to the lower number of embryos and proportionately heavier individual fetal weights in Siglec-6-OE mice, while control mice had a larger number of embryos and relatively lighter individual fetal weights. The findings from our mouse model collectively indicate that overexpression of Siglec-6, specifically in the placenta, plays a significant role in the pathogenesis of PE. However, the impact of GPR20 and NF-κB downstream of Siglec-6 on the development of PE remains to be elucidated.

## Conclusions

Our study revealed that Siglec-6 overexpression triggers mitochondrial dysfunction in trophoblasts through the SHP1/SHP2-NF-κB-GPR20 signaling pathway, which results in decreased trophoblast migration and invasion. Furthermore, our analysis of clinical samples indicates that the dysregulation of Siglec-6, NF-κB and GPR20 is associated with EO-PE. Overexpression of Siglec-6 specific to the placenta caused adverse placental mitochondrial dysfunction in pregnant mice and resulted in a PE-like syndrome. Thus, these findings suggest that Siglec-6 and GPR20 could serve as potential markers and targets for the clinical diagnosis and therapy of EO-PE.

### Electronic supplementary material

Below is the link to the electronic supplementary material.

Supplementary Material 1

## Data Availability

All relevant data and materials are freely available to any investigator on request.

## Abbreviations

ChIP: Chromatin immunoprecipitation
Co-IP: Co-Immunoprecipitation
DEG: Differentially expressed gene
EdU: 5-Ethynyl-2’-deoxyuridine
ELISA: Enzyme-linked immunosorbent assay
EO-PE: Early-onset preeclampsia
EVT: Extravillous trophoblast
FBS: Fetal bovine serum
H&E: Hematoxylin and eosin
IHC: Immunohistochemistry
ITIM: Immunoreceptor tyrosine-based inhibitory motif
LO-PE: Late-onset preeclampsia
MMP: Mitochondrial membrane potential
OCR: Oxygen consumption rate
PAS: Periodic Acid–Schiff
PE: Preeclampsia
qPCR: Real-time quantitative polymerase chain reaction
SEM: Standard error of the mean
Siglecs: Sialic acid-binding immunoglobulin-like lectins
SRC: Spare respiraiton capacity
TEM: Transmission electron microscopy
