# Supplementary material for: Upregulation of Siglec-6 induces mitochondrial dysfunction by promoting GPR20 expression in early-onset preeclampsia
Source: J Transl Med. 2024 Jul 22;22:674. doi: 10.1186/s12967-024-05505-z (PMC11265165; doi:10.1186/s12967-024-05505-z)
Supplement: Supplementary file 1 — Supplementary Material 1 [file 12967_2024_5505_MOESM1_ESM.docx]

**Supplementary Table 1**

Participant characteristics

|  | Normal  (n=15) | Perterm birth  (n=15) | EO-PE  (n=15) | * | # |
| --- | --- | --- | --- | --- | --- |
| Maternal Age | 30.3±3.1 | 30.5±3.8 | 31.2±5.0 | NS | NS |
| Gravity | 1.7±0.8 | 1.6±0.9 | 1.9±1.3 | NS | NS |
| Prarity | 1.0±0.6 | 1.1±0.4 | 0.9±0.5 | NS | NS |
| Progestational BMI | 23.4±2.4 | 23.1±1.6 | 24.2±3.2 | NS | NS |
| Delivery week | 38.8±0.5 | 34.4±1.0 | 34.7±0.5 | P＜0.0001 | NS |
| SBP_max_ (mmHg) | 116±11.7 | 126.3±8.6 | 155.1±12.0 | P＜0.0001 | P＜0.0001 |
| DBP_max_ (mmHg) | 75.5±8.6 | 77.5±10.8 | 98.7±0.9 | P＜0.0001 | P＜0.0001 |
| 24h proteinuria (g) | - | - | 2.0±0.9 | - | - |
| Infant Birthweight (g) | 3264.5±352.8 | 2335±438.1 | 2150.1±496.2 | P＜0.0001 | NS |

* Comparison of normal group with EO-PE group; # Comparison of preterm birth group with EO-PE group. All data are means ± SEM. One-way ANOVA followed by Tukey’s post-hoc test.

**Supplementary Table 2**

siRNAs sequences

| Genes | siRNA sequences |
| --- | --- |
| siSiglec-6#1 | 5’-CCUU CCAGCCU CG UACUAUTT-3’ |
|  | 5’-AUAGUACGAGGCUGGAAGGTT-3’ |
| siSiglec-6#2 | 5’-CCAGCUCAAUGUCUCCUAUTT-3’ |
|  | 5’-AUAGGAGACAUUGAGCUGGTT-3’ |
| siLeptin R#1 | 5’-GGAUAUUGGAGUAAUUGGATT-3’ |
|  | 5’-UCCAAUUACUCCAAUAUCCTT-3’ |
| siLeptin R#2 | 5’- CCUGGGCACAAGGACUUAAUUTT-3’ |
|  | 5’- AAUUAAGUCCUUGUGCCCAGGTT-3’ |

**Supplementary Table 3**

Primer sequences

| Genes | Primer sequences |
| --- | --- |
| Siglec-6 | Forward：5’-AGCCTCGTACTATGGTTATGGC-3’ |
|  | Reverse：5’-CACTTCTTCGTCTGGGTCGTT-3’ |
| GPR20 | Forward：5’-GCCCCTGTTCCACCTGTTT-3’ |
|  | Reverse：5’-CTACCAGTAGATCGGTCACCA-3’ |
| β-Actin | Forward：5’-CATGTACGTTGCTATCCAGGC-3’ |
|  | Reverse：5’-CTCCTTAATGTCACGCACGAT-3’ |
| GPR20 ChIP | Forward：5’- GGGACCGTCTCTGAGATTTCT-3’ |
|  | Reverse：5’- CTGAGTCACCGCACCAGT-3’ |

**Supplementary Table 4**

Antibodies information

| Antibodies | Manufacturers | Code |
| --- | --- | --- |
| Anti-SIGLEC-6 antibody | Abcam | Ab262851 |
| β-Actin antibody | CST | 3700 |
| Anti-HLA-G antibody | Santa Cruz | 21799 |
| Anti-Leptin Receptor antibody | Abcam | ab5593 |
| Parkin (Prk8) antibody | CST | 4211T |
| PINK1 (D8G3) Rabbit antibody | CST | 6946T |
| LC3B Rabbit antibody | ABclonal | A19665 |
| Anti-GPR20 antibody | Absin | Abs133109 |
| β-Actin antibody | CST | 3700 |
| Anti-PKA antibody | Abcam | ab76238 |
| Anti-SHP1 antibody | Abcam | ab227503 |
| Anti-SHP-2 antibody | CST | 3397S |
| DYKDDDDK Tag antibody | CST | 14793S |
| NF-κB p65 antibody | CST | 8242T |
| Phospho-NF-κB p65 antibody | CST | 3033T |
| ERK1/2 antibody | CST | 9695 |
| Phospho-ERK1/2 antibody | CST | 4370 |

**Figure S1**

**
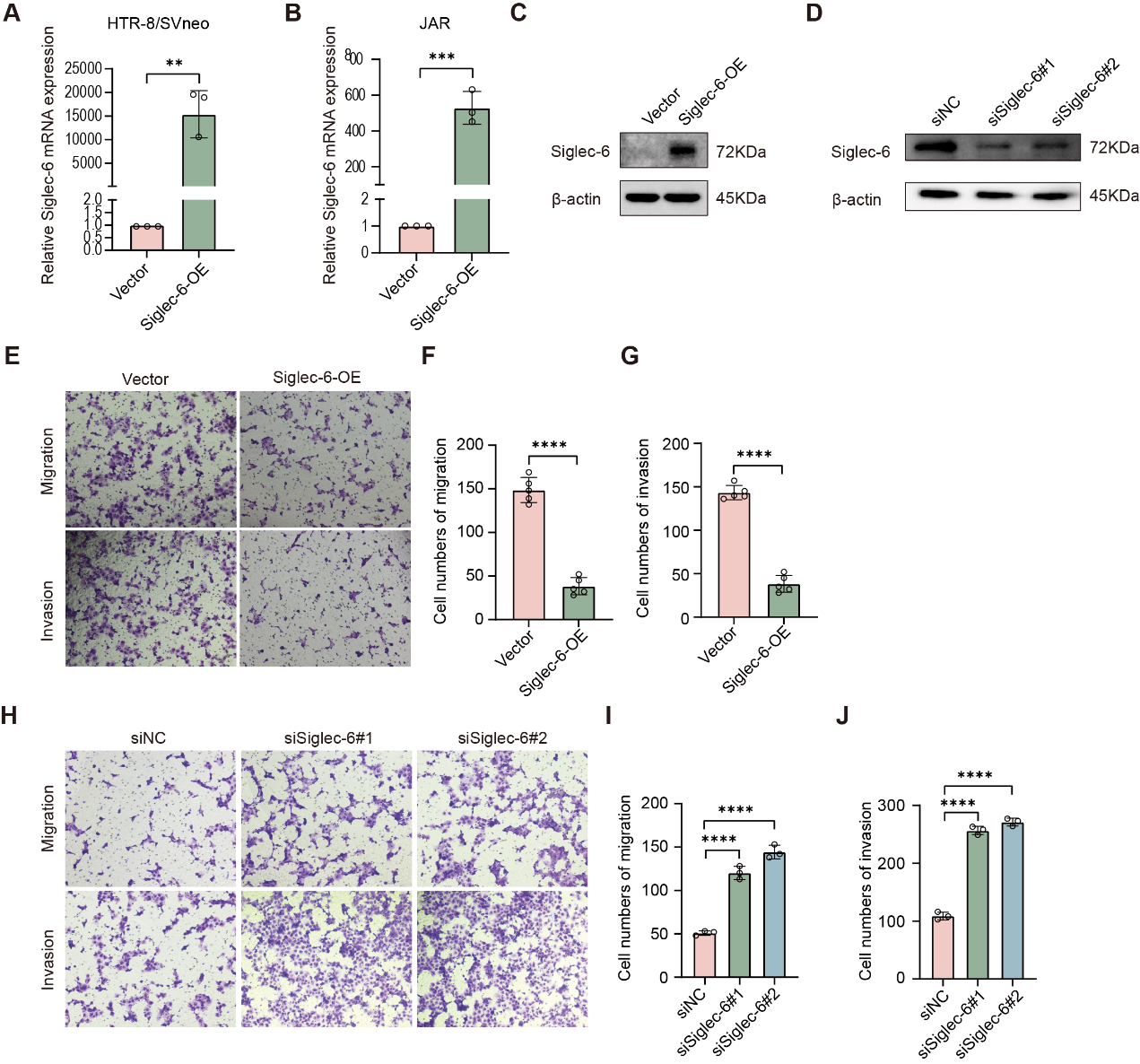
**

**Effects of Siglec-6 on trophoblast cell migration and invasion**

qRT-PCR analysis of the Siglec-6 mRNA levels in the stable Siglec-6 overexpressing HTR-8/SVneo (A) and JAR (B) cell lines. (C) Western blot analysis of Siglec-6 protein levels in the stable JAR cell line over-expressing Siglec-6. (D) Western blot analysis of the Siglec-6 protein level in JAR cells transfected with siNC or siSiglec-6. (E) Migration and invasion of stable JAR cell line over-expressing Siglec-6 were determined by transwell assay. Representative images are shown. (F, G) The number of migrated and invaded JAR cell line over-expressing Siglec-6 was counted. (H) Migration and invasion of JAR cells transfected with siNC or siSiglec-6 were determined by transwell assay. Representative images are shown. (I, J) The number of migrated and invaded JAR cells transfected with siNC or siSiglec-6 was counted. All the statistical data were analyzed by Student’s t-test (two groups) or one-way ANOVA (above two groups). All data are means ± SEM of three independent experiments performed in triplicate. ****p*＜0.001, *****p*＜0.0001.

**Figure S2**

**
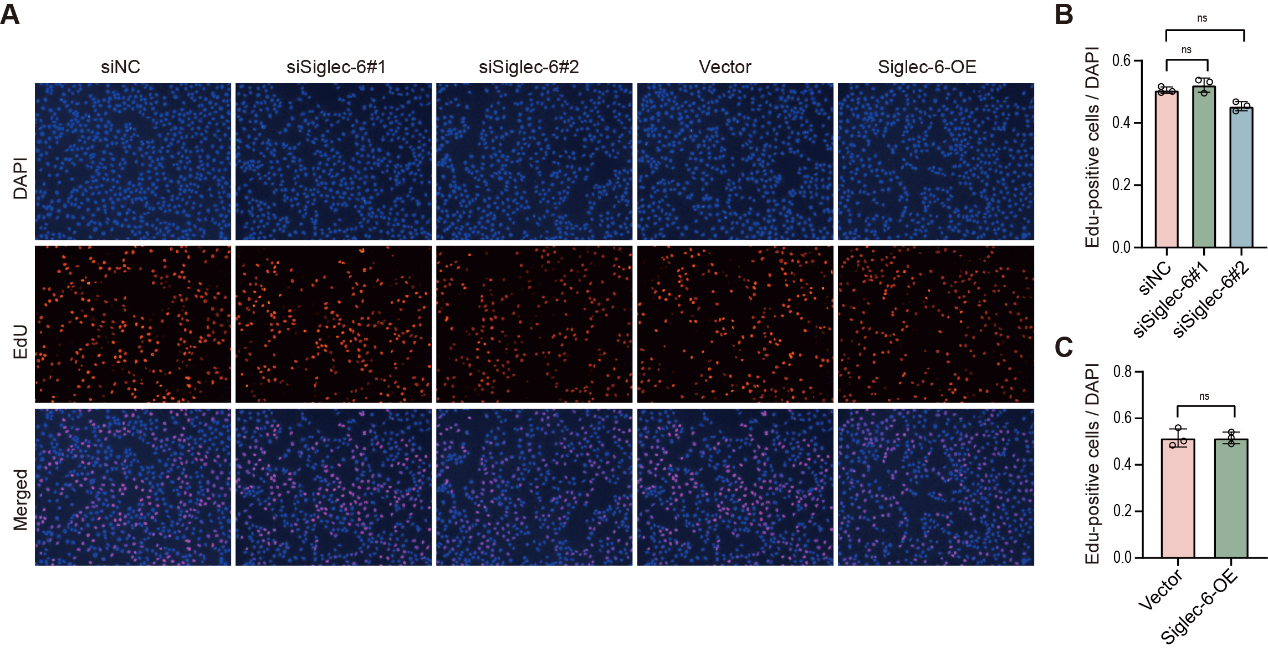
**

**Effects of Siglec-6 on trophoblast cell proliferation**

EdU (A) tests were carried out to evaluate the proliferation capacity of HTR-8/SVneo cells, and the EdU positivity rate was quantified (B, C). All the statistical data were analyzed by Student’s t-test (two groups) or one-way ANOVA (above two groups). All data are means ± SEM of three independent experiments performed in triplicate. ns, no significant difference.

**Figure S3**

**
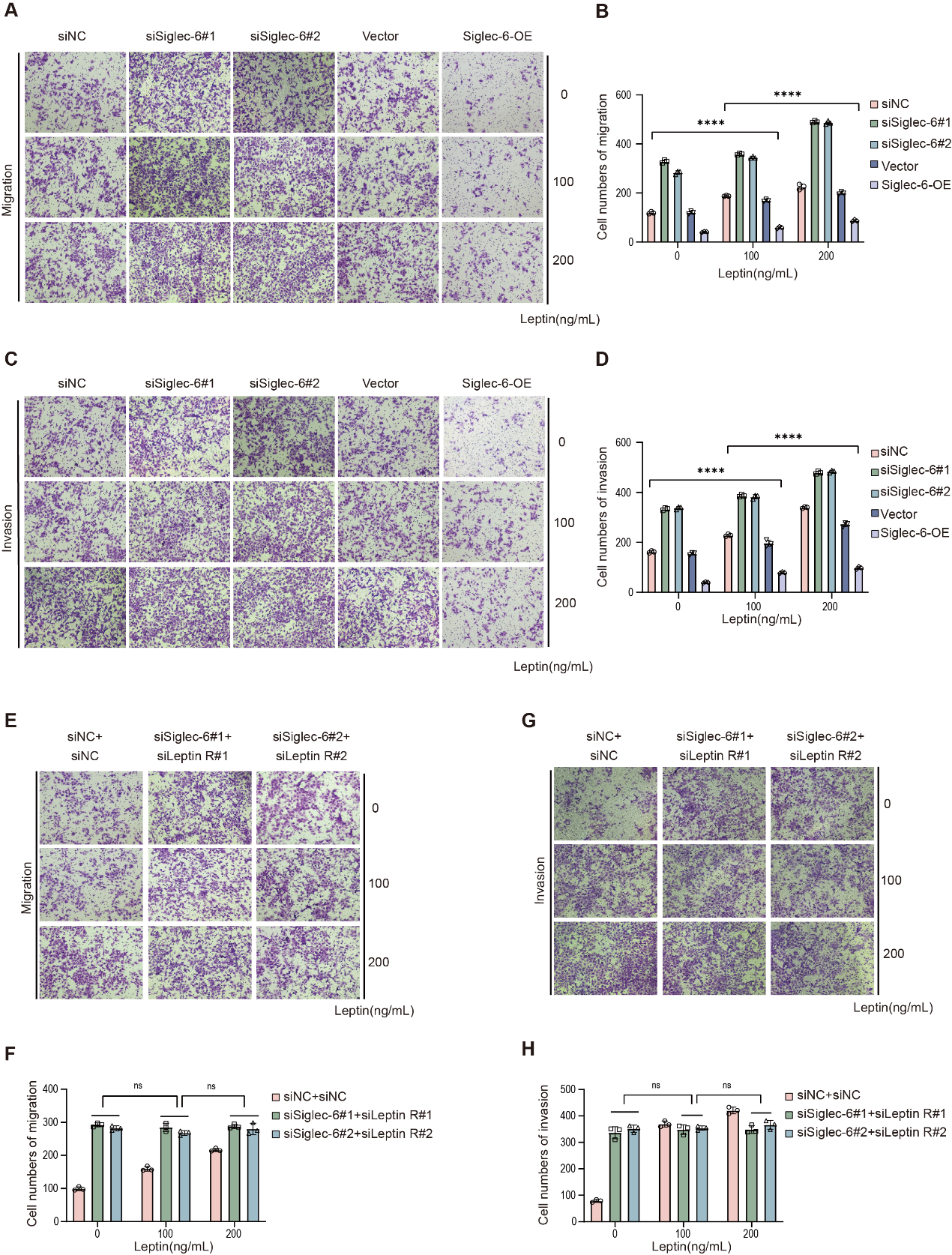
**

**Leptin affects trophoblast cell migration and invasion through leptin R but not Siglec-6**

(A-D) Representative images of the effects of overexpression or knockdown of Siglec-6 on HTR-8/SVneo cell migration and invasion at different concentrations of leptin (original magnification, 100×, scale bar=200 µm). (E-H) Representative images of the effects of knockdown of Siglec-6 and leptin R on HTR-8/SVneo cell migration and invasion at different concentrations of leptin. All the statistical data were analyzed by two-way ANOVA. All data are means ± SEM of three independent experiments performed in triplicate., *****p*＜0.0001, ns: no statistical difference.

**Figure S4**

**
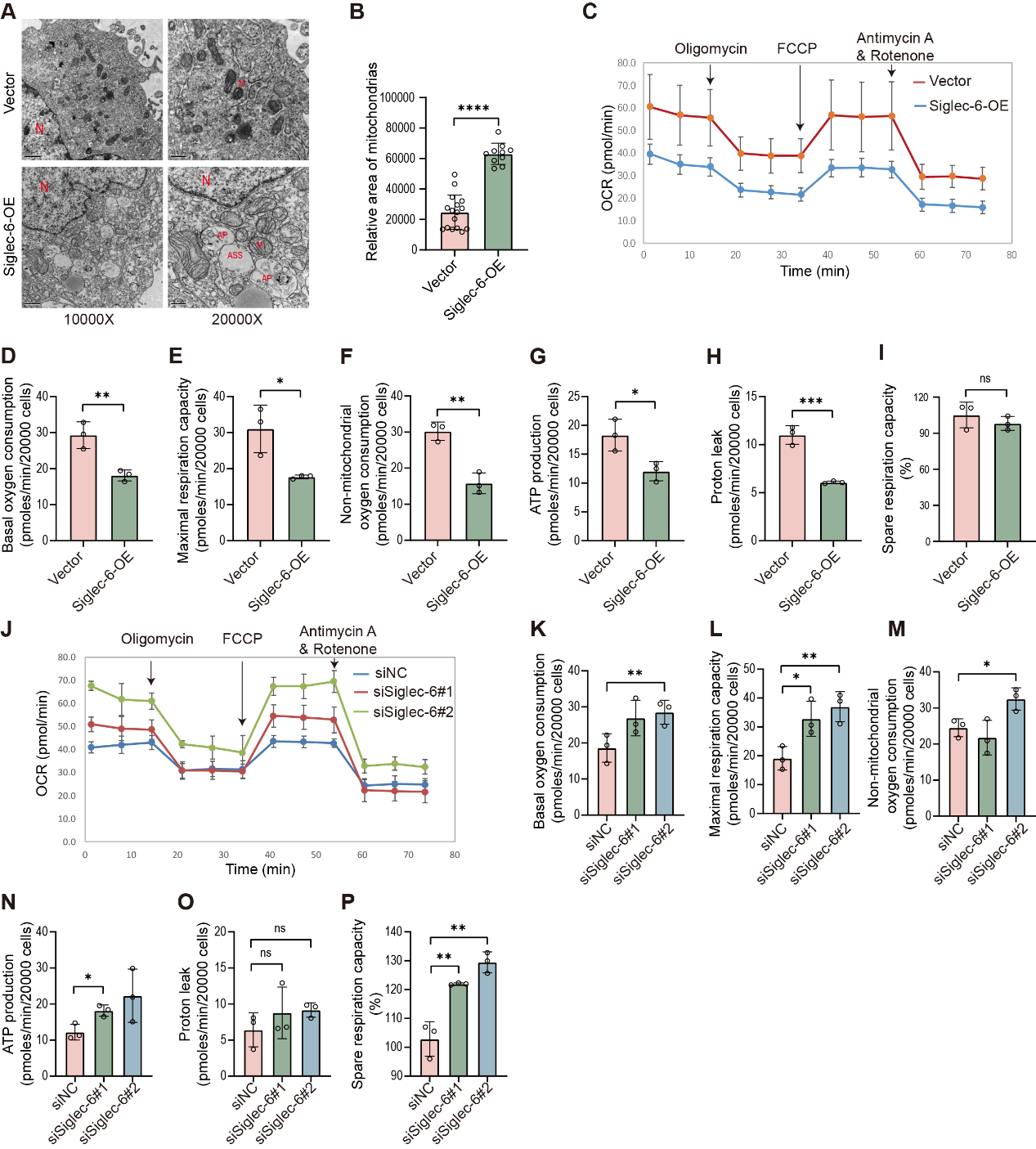
**

**Effects of Siglec-6 on mitochondrial morphology and function in JAR cells**

(A) TEM of the stable JAR cell line over-expressing Siglec-6. N, nucleus; M, mitochondria; ASS, autophagy lysosome; AP, autophagic vesicle. (TEM, original magnification, 10000×, scale bar=1 µm; 20000×, scale bar=0.5 µm). (B) Mitochondrial surface area was measured. (C, J) Mitochondrial oxygen consumption rate (OCR) was recorded using Seahorse analyzer. Basal oxygen consumption (D, K), maximal respiration capacity (E, L), non-mitochondrial oxygen consumption (F, M), spare respiratory capacity (G, N), proton leak (H, O) and ATP production (I, P) were determined by using the Seahorse analyzer in the stable JAR cell line over-expressing Siglec-6 and JAR cells transfected with siNC or siSiglec-6. Results are shown as mean ± SEM. All the statistical data were analyzed by Student’s t-test (B, D-I) or one-way ANOVA (K-P). **p*＜0.05, ***p*＜0.01, ns: no statistical difference.

**Figure S5**

**
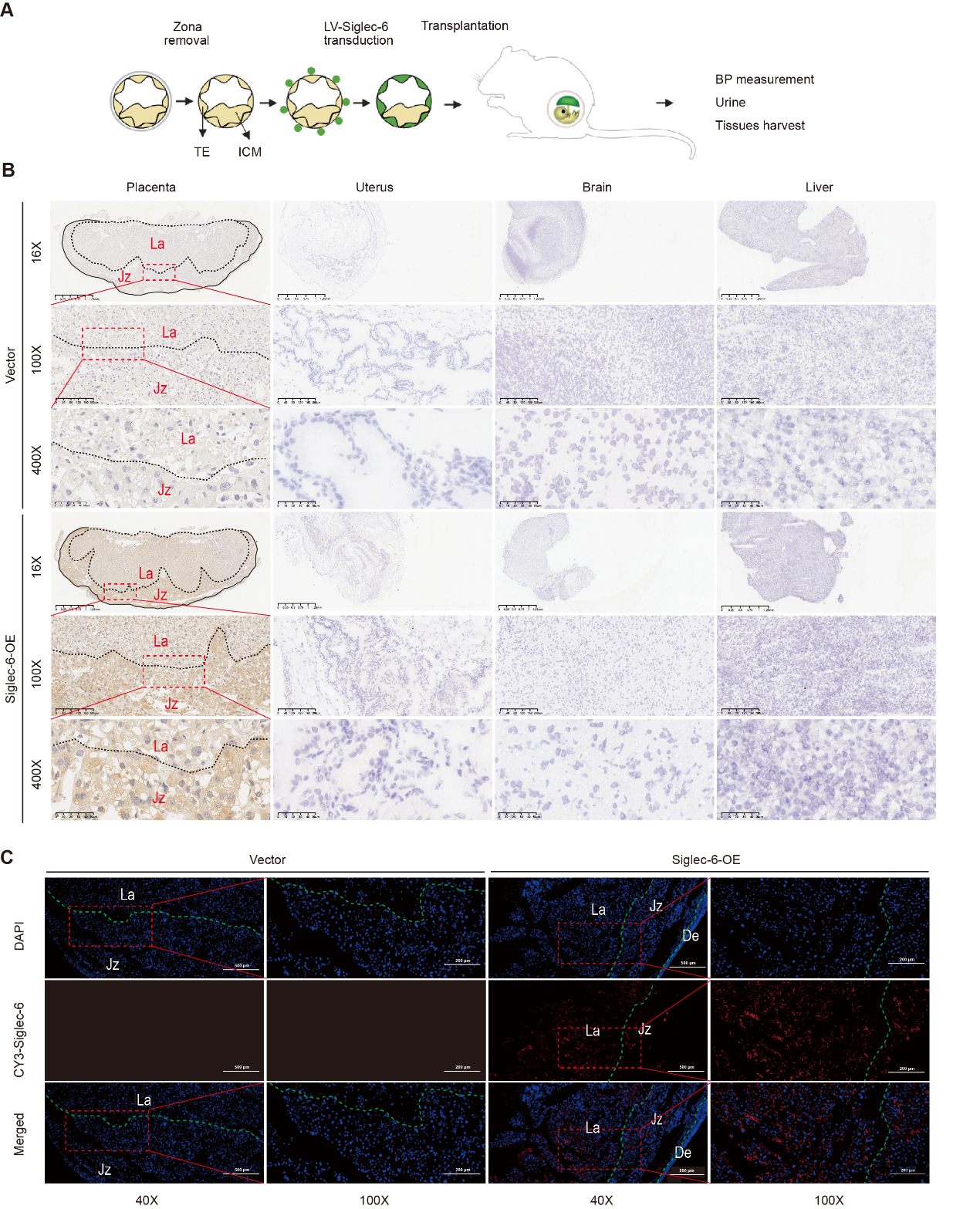
**

**Construction of a mouse model for placenta-specific overexpression of Siglec-6**

(A) Schematic diagram of experimental design. Lentiviral vectors that expressing the Siglec-6 gene were introduced into blastocysts with removed zona pellucida and implanted into pseudopregnant female mice. The gene-incorporated trophoblast ectodermal cell line forms the key element of the placenta and persistently expresses Siglec-6. TE, trophectoderm; ICM, inner cell mass. (B) Representative images demonstrate the expression of Siglec-6 in the placenta, uterus, brain and liver of mice within both the vector and Siglec-6 overexpression groups using IHC. (C) Representative images demonstrate the expression of Siglec-6 in the placenta of mice within both the vector and Siglec-6 overexpression groups using fluorescence in situ hybridization (original magnification, 16×, scale bar=1.25 mm; 40×, scale bar=500 µm ;100×, scale bar=200 µm; 400×, scale bar=50 µm). JZ, junctional zone; La, labyrinth; De, decidua.

**Figure S6**

**
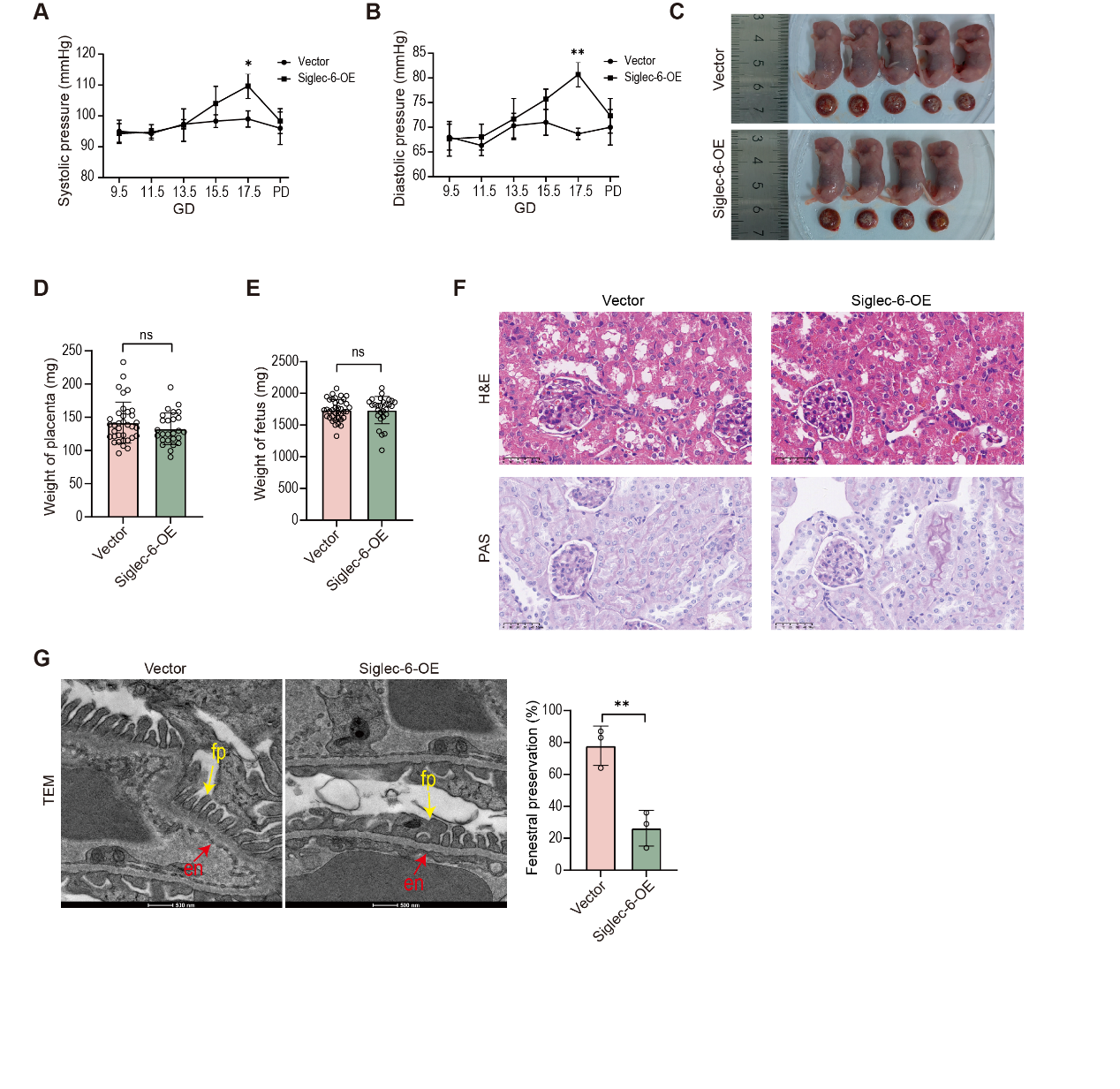
**

**Overexpression of Siglec-6 in the placenta specifically leads to a PE-like phenotype in pregnant mice**

Systolic blood pressure (A) and diastolic blood pressure (B) were measured in vector (n=4) and Siglec-6-OE (n=4) mice on gestational days 9.5, 11.5, 13.5, 15.5, 17.5, and postdelivery. Fetal morphology (C), birth weight (D), and placental weight (E) were evaluated to assess the impact of placenta-specific overexpression of Siglec-6. (F) H&E and PAS staining images show glomerular structures in vector and Siglec-6-OE mice, with glomerular diameter calculated. (G) TEM images of glomeruli from vector and Siglec-6-OE mice. fp, foot process; en, endothelial cells. Results are shown as mean ± SEM. All the statistical data were analyzed by Student’s t-test. **p*＜0.05, ***p*＜0.01, ns: no statistical difference.

**Figure S7**

**
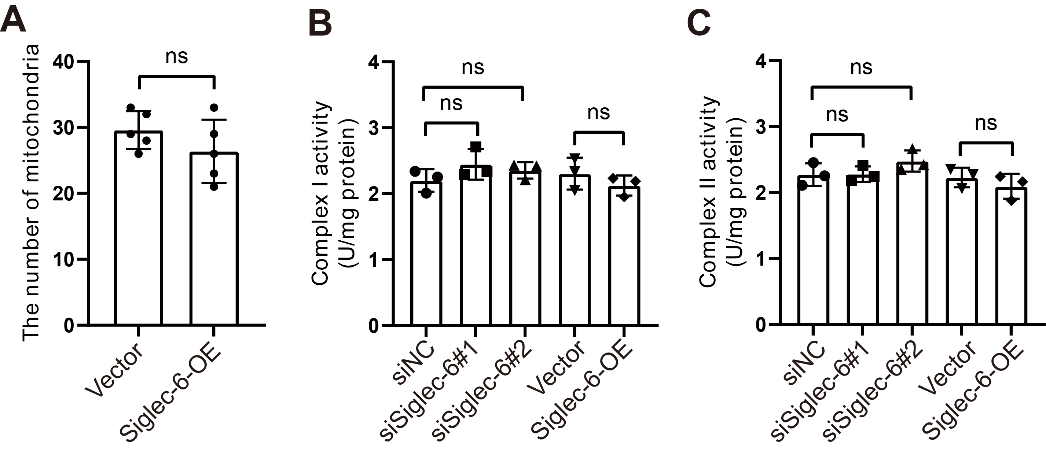
**

Effect of Siglec-6 on the number of mitochondria and activities of mitochondrial complex Ⅰ and complex Ⅱ in HTR-8/SVneo cells

(A) The number of mitochondria in stable HTR-8/SVneo cell line over-expressing Siglec-6 and control cells. The activities of mitochondrial complex Ⅰ (B) and Ⅱ (C) of the stable HTR-8/SVneo cell line over-expressing Siglec-6 and HTR-8/SVneo cells transfected with siNC or siSiglec-6 were measured.
